# Into the wild: a long-term study of begomovirus community dynamics in a natural landscape

**DOI:** 10.1099/jgv.0.002340

**Published:** 2026-10-12

**Authors:** João P. Herrera da Silva, César A. D. Xavier, Phedra G. S Oliveira, Márcio T. Godinho, Júlia B. Lage, José C. F. da Silva, Alison T. M. Lima, F. Murilo Zerbini

**Affiliations:** 1 Dep. de Fitopatologia/BIOAGRO, Universidade Federal de Viçosa, Viçosa, MG 36570-900, Brazil; 2 Horticultural Science Department, University of Florida, Gainesville, FL, USA; 3 Instituto de Ciências Agrárias, Universidade Federal de Uberlândia, Uberlândia, MG, Brazil

**Keywords:** geminivirus, Oxalis yellow vein virus (OxYVV), Sida yellow leaf curl virus (SiILCV), recombination, virus evolution

## Abstract

Begomoviruses have been the subject of many evolutionary studies that explored their temporal dynamics; however, most of the studies relied on sequences retrieved from public repositories rather than hypothesis-driven sampling. These datasets are often temporally or spatially unbalanced, which can bias molecular clock calibration, especially when the population exhibits strong genetic structure. Furthermore, most studies have focused on agricultural environments where anthropogenic activity may influence viral diversification. Longitudinal studies with systematic sampling in the natural environment are essential to understand begomovirus ecology and evolution. We monitored a begomovirus community over 12 years (2011–2014, 2016–2022) in a 300 m² Atlantic Forest regeneration area in southeastern Brazil, sampling 385 *Sida acuta* plants and obtaining 371 complete genomic segments (262 DNA-A and 109 DNA-B). We quantified community diversity and estimated substitution rates and demographic dynamics. Five begomoviruses were detected at the site, with Oxalis yellow vein virus (OxYVV; *Begomovirus oxalisflavi*) and Sida yellow leaf curl virus (SiYLCV; *Begomovirus sidaflavacontorsionis*) dominating the community. Species composition changed over time: OxYVV was the most frequent virus during the early years but was outnumbered by SiYLCV from 2016 onwards, with alternating prevalence in later years. Community diversity peaked in 2021 when all five viruses were detected co-occurring. The diversification of SiYLCV exhibited a higher relative contribution of recombination than mutation (ρ/θ>1), contrary to all other begomoviruses studied to date. Although we hypothesized that the changes in community composition observed in 2016 resulted from a genetic bottleneck in the OxYVV population, no evidence of such an event was detected. Instead, we observed a demographic expansion of SiYLCV, consistent with its increase in frequency. This study reveals the complex community dynamics of begomoviruses in natural landscapes and underscores the role of wild plants as reservoirs of viral diversity, providing a baseline for comparisons with agroecosystems to inform management strategies.

## Data Availability

All sequences obtained in this study have been deposited in GenBank. Accession numbers are listed in Table S1 (available in the online Supplementary Material).

## Introduction

 It has been estimated that, over the last century, more than half of emerging diseases in plants, animals and humans have been of viral origin [1–4]. Ecosystem degradation, the introduction of exotic organisms and rapid viral evolutionary dynamics have been identified as major drivers of viral disease emergence [1, 5–8]. Plant viruses represent a threat to food security and to the conservation of natural ecosystems. Historically, studies exploring viral population dynamics have been conducted mainly in agroecosystem contexts [9, 10]. However, this scenario has changed in the 21st century, and studies investigating viral diversity in non-cultivated plants have become more frequent [11–14]. However, most of these studies represent spatial and temporal snapshots, and the long-term dynamics of viral communities in natural ecosystems remain largely underexplored [15, 16].

Non-cultivated plants have been recognized as important reservoirs of viral biodiversity [8, 11, 17–20]. Many of these plants, when not managed, can persist in the field for long periods of time, acting as sources of inoculum for cultivated plants [21, 22]. In addition, some plant species are considered true ‘mixing vessels’, as they can host numerous viral species, thereby creating unique opportunities for coinfections [1, 23, 24]. The frequent occurrence of coinfections creates opportunities for recombination events, potentially contributing to viral diversification at both micro- and macroevolutionary scales and leading to the emergence of novel viral genotypes or virus species, respectively [25–27].

Begomoviruses are recognized for causing devastating epidemics in plants across the world, particularly in tropical and subtropical regions [28]. They are transmitted by insect vectors from the *Bemisia tabaci* cryptic species complex (whiteflies). Begomoviruses have small ssDNA genomes of ~2.6 kb, which may be mono- or bipartite [29]. Despite their DNA genomes, begomoviruses exhibit evolutionary rates comparable to those of ssRNA viruses, driven by high substitution rates and frequent recombination events [30–32].

In Brazil, the emergence of begomoviruses in tomatoes during the mid-1990s has been linked to the introduction of the highly polyphagous *Bemisia tabaci* MEAM1 (formerly biotype B), which facilitated the spillover of multiple begomoviruses from indigenous non-cultivated hosts into crop species [33]. A similar scenario has been proposed for the begomovirus epidemics in cassava in Africa during the 1980s and 1990s. Cassava is native to South America and, interestingly, begomovirus infections have never been documented in its region of origin, suggesting that the viruses responsible for these epidemics were likely acquired through lateral transmission from indigenous African plants [34].

The temporal dynamics of begomovirus populations have been addressed in a number of studies that have expanded our understanding of the processes that contribute to the differentiation of these populations over time, as well as estimated their evolutionary rates [30, 31, 35–38]. However, as mentioned above, all these studies were carried out under an agricultural context spanning large areas, with some specific locations and/or time points being overrepresented in relation to others. In addition, many of these studies made use of data from GenBank, whose sampling strategies are not hypothesis-driven and would therefore be subject to the introduction of biases and distortions in the estimation of evolutionary rates, as they do not take into account the genetic structure of populations. Previous studies have demonstrated that population genetic structure can affect the pace of the molecular clock due to the asymmetric accumulation of neutral mutations in different populations [39]. The absence of longitudinal studies based on systematic sampling over time within the same area, particularly in environments with low or no anthropogenic activity, represents a critical limitation for understanding the long-term evolution and ecological dynamics of begomoviruses.

Here, we conducted a longitudinal study based on annual sampling of the non-cultivated host *Sida acuta* (Malvaceae) over more than a decade in a natural landscape characterized by low anthropogenic disturbance. Our objective was to assess how begomovirus population diversity and variability are structured over time within a small, non-agricultural environment and to evaluate whether these local patterns reflect those observed at broader spatial scales. This study represents the first systematic effort to characterize the temporal dynamics of begomoviruses in natural landscapes.

## Methods

### Data sampling

*S. acuta* plants (Malvaceae) displaying symptoms of begomovirus infection (Fig. S1, available in the online Supplementary Material) were collected annually in early December, at the same location, over a 12-year period (2011-14, 2016-22). Plant species were identified based on morphological characteristics using dichotomous taxonomic keys [40]. The sampling area, with ~300 m², is located in a transition zone between Atlantic Forest remnants and a suburban area outside of an agricultural context, with minimal human intervention, near Viçosa, Minas Gerais, Brazil (20º 45’ S, 42º 52’ W; Fig. S2). Whiteflies were only sporadically present in the area. Sampling was conducted randomly across the study area, and only symptomatic plants were collected (based on visual estimations, 50–80% of the plants had symptoms in a given year). On average, 35 plants were collected per year, totalling 360 plants over the study period. Each specimen was identified, photographed, press-dried and stored at room temperature in the Laboratory of Virus Ecology and Evolution at UFV. For each plant, the youngest fully expanded leaf was collected for DNA extraction.

### Cloning and sequencing

Total DNA was extracted from all plant samples following the protocol by Doyle and Doyle [41]. For whole-genome amplification via rolling circle amplification (RCA), ~1 ng of DNA was used as a template [42]. RCA products were digested with restriction enzymes to yield linear, genomic-length molecules (~2,600 bp), which were subsequently ligated into the pBluescript KS+plasmid vector (Agilent Technologies), previously digested with the same restriction enzyme and dephosphorylated. Recombinant plasmids were introduced into *Escherichia coli* DH5α competent cells by electroporation [43]. Positive clones were fully Sanger-sequenced at Macrogen (Seoul, South Korea) using a primer-walking strategy.

### Phylogenetic analysis

Contigs were assembled using Geneious software [44] and oriented to start at the cleavage site of the replication origin (TAATATT//AC), following the convention established for begomoviruses [29]. An initial blastn search against the GenBank nt database [45] was performed to determine the viruses closest to the analysed sequences. Taxonomic assignments were carried out using Sequence Demarcation Tool v1.2, according to the guidelines of the *Geminiviridae* Study Group of the ICTV [46]. Begomovirus isolates with >91% pairwise identity correspond to the same species, and isolates from the same species with >94% identity are considered as belonging to the same strain [46]. Genetic content and genomic organization analyses were carried out using ORFfinder [47].

Multiple sequence alignments were constructed for the DNA-A and DNA-B as well as each of the ORFs encoded by each component with MAFFT v7 [48], using default parameters. Maximum likelihood phylogenetic analysis was performed using RaxML-NG [49]. The most suitable evolutionary model was determined using ModelTest-NG based on the Akaike Information Criterion [50]. The analyses were performed using 1,000 bootstrap replicates.

### Assessment of begomovirus diversity over time

Diversity comparisons between samples collected on different years were based on the effective number of species (Hill numbers) [51] using the iNEXT package of R software [52]. This metric allows the quantification of the species richness of a system, as well as evenness. Diversity orders are estimated using the scaling parameter q [53]. Diversity of order zero (q=0) corresponds to species richness and gives equal weight to all detected species, regardless of their frequency. A value of q=1 corresponds to the exponential of Shannon entropy and weights species according to their relative frequencies, while q=2 corresponds to the inverse Simpson diversity and gives greater weight to dominant species [53, 54]. We defined our input data as the number of DNA-A sequences assigned to each begomovirus species per sampling year, normalized by the number of plants analysed each year. Since the number of plants analysed was not the same each year, we performed rarefaction and extrapolation analyses using iNEXT to evaluate whether the sample size would have been sufficient to capture viral diversity [52].

In addition to the conventional approach for estimating Hill numbers, we monitored the temporal trajectory of species that make up the viral community using a recently developed approach named KHILL [55]. The basis of the tool is the same, but it is not based on counting to calculate diversity. Instead, this strategy is based on the diversity of information contained in k-mer libraries obtained from genomic data. KHILL performs comparisons between unaligned substrings of length k, thus the containers are considered as libraries of k-mers. The sets of k-mers were determined based on complete sequences, using sliding windows of k=19 (the programme’s default). betaEnt is a function of the sum of the contribution of each individual genome to total entropy, which in turn is given by the frequency at which this k-mer is represented within the sample. Total entropy is then used to determine KHILL, which is given by exp(betaEnt), providing a measure of diversity [55]. The amount of information is thus accumulated, allowing for comparisons between containers based on the KHILL curve. Very homogeneous data sets (with high information overlap) will produce a KHILL equal to 1, while complex data sets without information overlap will generate a higher KHILL number. The metric takes relative abundance into account, as populations with several species distributed in uniform proportions will produce higher KHILL values than populations where one species prevails. This method is free from sequence alignment and tree construction. As in the previously described approach, our containers were defined based on the sampling years.

To evaluate whether the temporal patterns detected by KHILL were also reflected in explicit phylogenetic diversity, we performed a complementary Faith’s phylogenetic diversity analysis [56]. For this analysis, we used the DNA-A phylogeny to build a presence/absence community matrix with sampling years as assemblages and tree tips as terminal taxa. Faith’s phylogenetic diversity was estimated using the pd function in the picante package [57], using branch lengths from the rooted phylogeny. Because Faith’s phylogenetic diversity reflects the total branch length represented by sampled taxa and can increase with sampling depth [56], we evaluated its relationship with the number of sequences sampled per year and generated rarefied estimates standardized to the minimum yearly sequence count.

### Estimates of genetic variability

We calculated the average nt diversity index, π [58], for both genomic segments and for each gene. Estimates of π values were computed through pairwise comparisons using a Python script developed by Lima *et al*. [25]. We computed a 95% confidence interval for the mean values of π through a bootstrap test with 1,000 non-parametric simulations using the boot package in R (https://cran.r-project.org/web/packages/boot/boot.pdf). Additional variability indices were computed using DnaSP v6 [59]. Using phylogenetic methods, we inferred mutational biases in the complete genome datasets of each species. The number of nt substitutions was estimated by mapping base changes onto the phylogenetic tree of each species using parsimony methods implemented in PAUP* [60]. We then compared the deviations of the observed frequencies of each substitution type with their expected values through χ² significance tests.

### Recombination analysis

To investigate the occurrence of recombination events, multiple sequence alignments of complete DNA-A and DNA-B segments were scanned separately using the seven methods implemented in the RDP5 package [61], using default parameters. Statistical significance was inferred by *P*-values lower than a Bonferroni-corrected *α* = 0.05 cutoff. As recommended by the authors, only recombination events detected by at least four methods were considered reliable.

Phylogenetic networks were built with the aim of capturing phylogenetic inconsistencies caused by hybridization. The networks were inferred using the Neighbour-Net algorithm implemented in SplitsTree v4.19.2 [62].

To investigate recombination events on a fine scale, recombination rates were inferred throughout the genome. Watterson’s estimator of the mutation rate (θ_W_) was calculated using the pairwise package in LDHat [63]. Furthermore, we estimated the relative contribution of mutation and recombination to the diversification of the populations by calculating the population-scaled ratio between recombination and mutation rates (ρ/θ_W_). Some adjustments needed to be done to the Oxalis yellow vein virus (OxYVV) data set, since the likelihood lookup table available with θ=0.001 goes up to only 120 sequences, and the OxYVV data set exceeded this value. As the computational cost to generate a likelihood lookup table is high, we adjusted the data set using CD-Hit [64], eliminating sequences with >99.96% identity and thus reducing the data set to 99 sequences.

### Temporal signal evaluation and demographic analysis

The temporal signal strength was evaluated using Bayesian evaluation of temporal signal (BETS) [65] implemented in BEAST v1.10.4. Two scenarios were considered. In the first one, the data was treated as being isochronous. In the second one, we treated the data as heterochronous [66], estimated the marginal likelihood using generalized stepping-stone sampling [67], and then computed the Bayes factor [68]. The molecular clock that best suited the data was also determined based on the Bayes factor, with a Markov chain Monte Carlo (MCMC) chain length of 2×10^8^ with 10% burn-in, and a number of stepping stones of 100, with an MCMC length of 2×10^6^. The convergence of the analysis was assessed using Tracer v1.7 and was determined based on the effective sample size, which should assume values of at least 200, and by the degree of interdependence of the samples assessed based on the degree of mixing of the parameters [69]. The time scale trees were summarized using the Maximum Clade Credibility (MCC) method in TreeAnnotaor v2.6.4 [70]. The substitution/site/year rates and time to the most recent common ancestor (TMRCA) were estimated with a 95% highest posterior density (HPD) confidence interval. The trajectory of populations over time was monitored through the construction of Bayesian Skygrid coalescent models [71].

### Selection analysis

Prior to selection analyses, a search for recombination breakpoints was carried out for each data set using the Genetic Algorithm for Recombination Detection (GARD) [72], and sequences identified as recombinant were excluded from the corresponding data sets. Selection analysis was performed for each of the coding regions of the genome of each species, using three methods: Single Likelihood Ancestor Counting (SLAC) [73], Mixed Effects Model of Evolution (MEME) [74] and Fast Unconstrained Bayesian AppRoximation (FUBAR) [75], all implemented in the DataMonkey webserver [76]. Relationships between synonymous and non-synonymous substitution rates (ω) were calculated using SLAC. We also performed Tajima’s D neutrality tests and calculated Fu and Li’s D* and F* statistics in DnaSP v6 [59].

## Results

### *S. acuta* is a ‘mixing vessel’ for begomoviruses

Over the course of 12 years, we collected 385 *S. acuta* plants with symptoms of begomovirus infection (Fig. S1) in the same small area (300 m^2^) near Viçosa, MG (Fig. S2). A total of 371 clones corresponding to full-length begomovirus components were obtained from 241 individual plant samples, 262 corresponding to a DNA-A and 109 corresponding to a DNA-B (Table S1). Because begomovirus species demarcation is based on DNA-A nt identity percentages [29], we prioritized sequencing this genomic component for the purposes of this study; when possible, we recovered DNA-B from a subset of samples. Maximum likelihood phylogenetic trees were reconstructed to assess the taxonomic placement of the isolates; since species demarcation criteria are based on the complete genomic segment, recombinant blocks were not removed in this specific analysis.

In total, we detected five bipartite begomoviruses (Tables S1 and S2). The most abundant was OxYVV (*Begomovirus oxalisflavi*), from which we obtained 162 DNA-A (127 haplotypes) and 52 DNA-B clones (40 haplotypes), followed by Sida yellow leaf curl virus (SiYLCV; *Begomovirus sidaflavacontorsionis*), which accounted for 77 DNA-A (59 haplotypes) and 44 DNA-B clones (35 haplotypes), Sida micrantha mosaic virus (SimMV; *Begomovirus sidamicranthae*) with 17 DNA-A (10 haplotypes) and 9 DNA-B (6 haplotypes) clones, Sida mottle virus (SiMV; *Begomovirus sidavariati*) with 5 DNA-A and 4 DNA-B clones, and a single DNA-A clone of Macroptilium yellow vein virus (MaYVV; *Begomovirus macroptilivenae*). Mixed infections were detected in a small fraction of plants, representing 6% of the total sampled individuals (Table S3).

Comparison of the DNA-A sequences indicated that the OxYVV population comprises two strains, S1 and S2, with isolates from each strain sharing a maximum DNA-A identity of 93.8% with those from the other strain (Fig. S3). The S1 strain is much more prevalent, totalling 155 clones and can be subdivided into five variants (S1a–e), with pairwise identity percentages above the strain threshold (95–97% among variants; Fig. S3). The remaining seven OxYVV clones correspond to the S2 strain. For SiYLCV, the population is comprised of a single variant, with 97.5–98% identities among all clones (Fig. S4). The SimMV population harbours two highly divergent variants that are very close to the strain demarcation threshold (93.8–94.5% identity between clones of the two variants; Fig. S5).

The phylogenetic tree inferred from the complete DNA-A sequences showed four well-supported clades and one long branch, each corresponding to one of the species mentioned above (Fig. 1a). The clade corresponding to OxYVV branches into six subclades corresponding to the five variants of strain S1 and the one group of strain S2 sequences. The bootstrap values of some of the branches that separate the S1 variants are low, possibly due to the high identity values between individual sequences. The clade that harbours SiYLCV also presents several subclades but with short genetic distances, consistent with the classification of all isolates as a single variant (Fig. 1a).

**Fig. 1.**
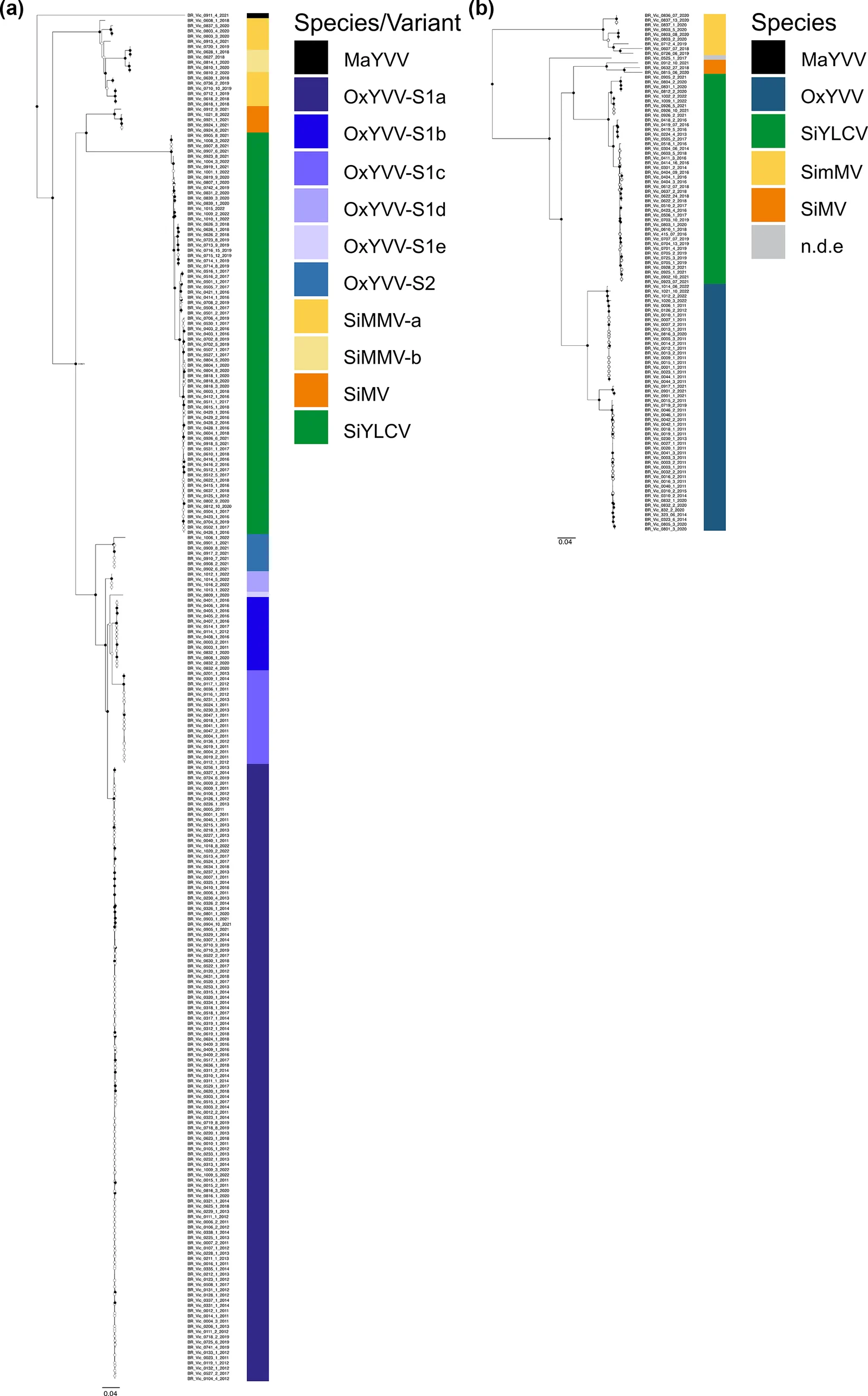
Phylogenetic trees based on the complete DNA-A (**a**) and DNA-B (**b**) nt sequences of the begomovirus isolates obtained in this work. Nodes to the right of branches with bootstrap support (1,000 replications) equal to or higher than 75% are indicated by black circles, and those with values lower than 75% by white circles. Isolates classified as members of the species OxYVV are represented in blue (or different shades of blue for the DNA-A tree), SiYLCV in green, SimMV in yellow, SiMV in orange and MaYVV in black. An isolate for which species assignment was not possible is represented in grey in the DNA-B tree. Species, strains and variants were identified based on the demarcation criteria established by the *Geminiviridae* Study Group of the ICTV [46]. The scale bar represents nt substitutions per site.

The phylogenetic tree based on the complete DNA-B sequences also formed four large clades corresponding to the four most prevalent species (Fig. 1b). The clade corresponding to OxYVV is subdivided into three well-supported subclades. A second clade corresponding to SiYLCV is subdivided into four subclades, all well supported, but with shorter genetic distances when compared to the subclades formed by OxYVV isolates (Fig. 1b). The tree also has a long, distinctive branch corresponding to a DNA-B sequence that probably represents a new species. However, since a cognate DNA-A (on which taxonomy is based [46]) was not cloned, it was not possible to confirm the identity of this isolate.

Together, these results indicate that the *S. acuta* plants harbour a complex begomovirus community composed of at least five viruses, some of which can be divided into strains and variants. Taking into consideration the small size of the sampling area, these results demonstrate how non-cultivated hosts may constitute sources of viral diversity.

### Temporal dynamics reveal fluctuations in the frequencies of viruses and their variants in the landscape

The temporal dynamics of begomoviruses in the community were characterized based on the distribution of the relative frequencies of DNA-A for each species, strain and variant, given that this genomic component underpins the definition of these groups. Major changes were observed in the composition of species and variants over the years (Fig. 2; Table S2). OxYVV was prevalent from 2011 to 2014, with three variants present (OxYVV-S1a, S1b and S1c). OxYVV-S1a was at a higher frequency in the first year and its frequency increased until 2014. OxYVV-S1b had a lower frequency during this period and was not detected in 2013 and 2014. OxYVV-S1c appeared in second place in terms of frequency, but its incidence was reduced in subsequent years. In 2012, a single plant infected with SiYLCV was detected for the first time. The year 2015 was a gap in our sampling. In 2016, there was a major change in the proportion of species and variants present at the site, with SiYLCV outnumbering OxYVV as the prevalent virus. Another change was that OxYVV-S1b was detected again and this time at a higher frequency than OxYVV-S1a. In 2017, the frequency of OxYVV-S1a increased again, while OxYVV-S1b and SiYLCV had a slight reduction. In 2018, we observed the local emergence of SimMV, with OxYVV-S1 and SiYLCV exhibiting balanced frequencies between themselves. In the three subsequent years, there were fluctuations in the frequencies of these three viruses, but always with an alternation between OxYVV and SiYLCV in terms of prevalence. The variant OxYVV-S1e emerged in 2020. The year 2021 marked the first detection of MaYVV and SiMV, in addition to the OxYVV-S2 strain. In 2022, we detected the two strains of OxYVV and the emergence of OxYVV-S1d (Fig. 2b).

**Fig. 2.**
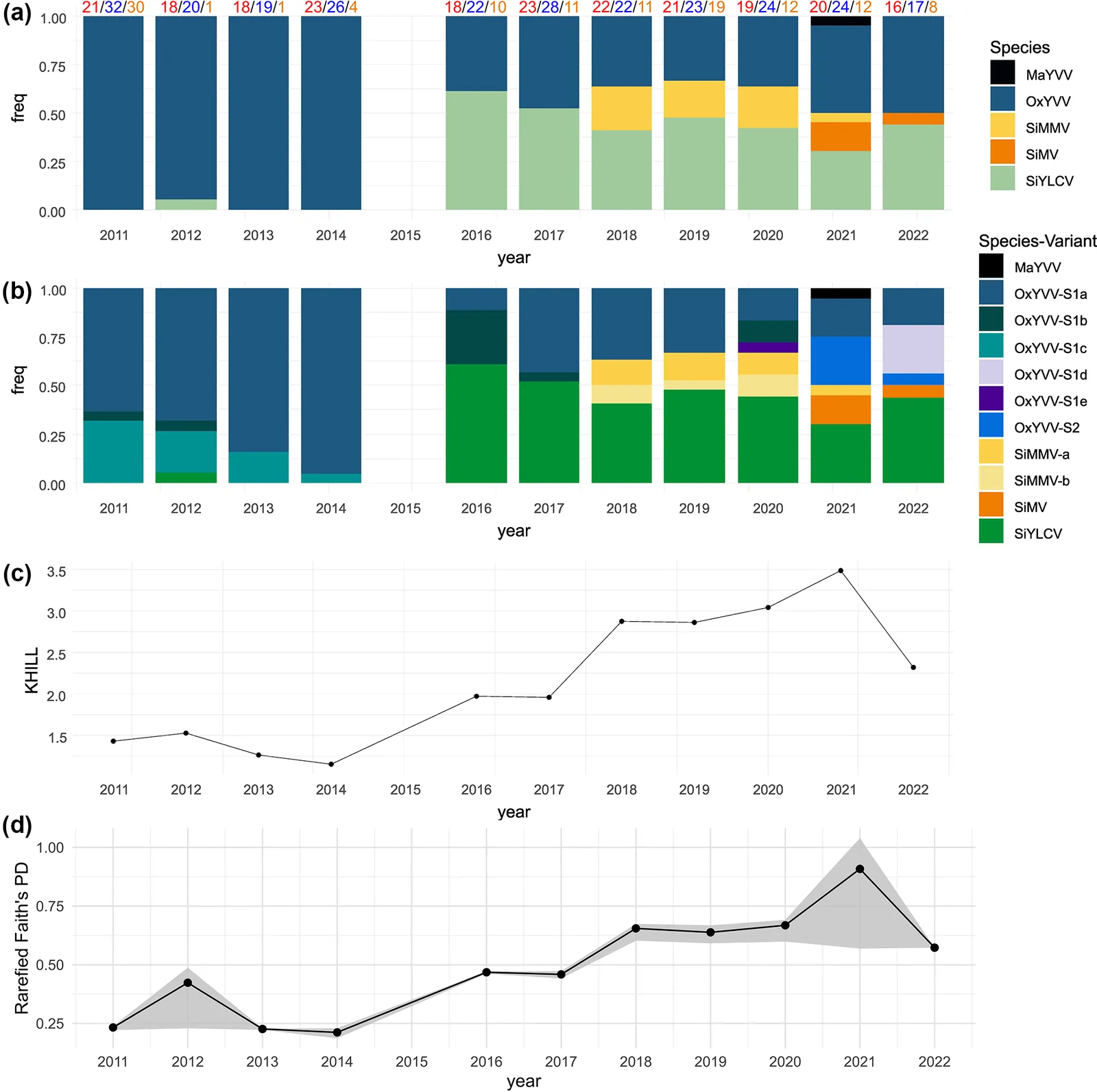
(a) Proportion of individuals of each begomovirus sampled in each year. The data is based on the sum of DNA-A and DNA-B sequences obtained each year. The colour scale on the right indicates species, strain and variant assignments. The numbers at the top of the bars indicate the number of plants analysed (red), the number of DNA-A sequences recovered (blue) and the number of DNA-B sequences (orange). (**b)** Relative proportion of each species and their respective variants isolated in each year. The data is based on DNA-A sequences only, since there are no criteria for strain and variant assignment based on the DNA-B. (**c)** KHILL values, which are estimated based on the entropy and represent the dynamics of the landscape over the years. (**d)** Rarefied Faith’s phylogenetic diversity estimated for DNA-A sequences in each sampling year, standardized to the minimum yearly sequence count. The shaded area represents the 95% interval generated from rarefaction replicates.

Hill numbers were calculated to assess diversity over time. This strategy entails a simple and intuitive measure that allows us to compare the diversity of species that make up a community. We opted to use species counts to assess diversity; another option would be to use variant diversity, but this could make our interpretation confusing (see below). The years 2011, 2013 and 2014 had only one species detected (Fig. 2a), in which case the value converged to unity (Fig. S6). The years 2012, 2016 and 2017 also had very low diversity, and the overlap of the three orders of diversity in 2016 and 2017 indicates that two species were represented in similar proportions in those years (Fig. S6). In 2018, 2019, 2020 and 2022, the richness values were equal to 3, and in 2021, it was equal to 5, the highest value observed in our time series. There was no significant difference between diversity orders 1 and 2 from 2018 to 2022 (Fig. S6). The effective number of species approached richness in 2016, 2017, 2018 and 2020, indicating that in these years the community was dominated by equally abundant species. Sample-size-based rarefaction and extrapolation curves (Fig. S6) further illustrate this temporal pattern, showing that community diversity remained consistently low between 2011 and 2017, followed by a marked increase from 2018 onwards, with 2021 exhibiting the highest estimated species richness in the time series.

To capture variations in diversity on a finer scale, we used KHILL, an approach that is also based on Hill numbers but uses DNA sequences and calculates the degree of entropy. We noticed changes in the behaviour of the KHILL curve that followed the dynamics of diversity changes over time (Fig. 2c). The KHILL curve was able to effectively capture changes in the diversity of species present at the site and beyond, detecting nuances in the dynamics of variants within the community. This can be observed in the first 4 years, as the KHILL values decrease together with the reduction in incidence of minor variants of OxYVV (Fig. 2c). This was followed by a sudden increase starting in 2016 until it peaked in 2021, when five species were detected. A similar temporal trend was also recovered by the rarefied Faith’s phylogenetic diversity analysis (Fig. 2d), indicating that the KHILL-based increase in genomic information diversity was accompanied by greater phylogenetic breadth among sampled DNA-A sequences.

### Genetic variability of begomoviruses in *S. acuta*

The genetic variability of each virus was inferred separately. Due to the size of the data sets, we did these estimates only for three of the five viruses detected in the area (OxYVV, SiYLCV and SimMV). The other two viruses (SiMV and MaYVV) were represented by a very small number of sequences (5 and 1, respectively). We estimated the variability for each genomic component and also for their genes (Table 1). In general, the DNA-B showed greater variability compared to the DNA-A, as has been reported for most bipartite begomoviruses [77, 78]. For both components, SimMV was the virus that showed the greatest variability, followed by OxYVV and SiYLCV (Table 1). When we looked at each gene separately, *Rep* and *Ren* showed the greatest variability. For the DNA-B, the *MP* genes of SimMV and OxYVV showed greater variability, while for SiYLCV the *NSP* gene had higher variability (Table 1).

**Table 1.** Genetic variability indices of the begomoviruses infecting *S. acuta* in Viçosa, MG

Species	No. of sequence	No. of haplotype (h)	Haplotype diversity (Hd)	Nt diversity (π)		
**OxYVV**				**Mean**	**CI lower**	**CI upper**
DNA-A	162	127	0.993	0.0197	0.0194	0.02
DNA-B	52	40	0.986	0.0479	0.0456	0.0502
*CP*	162	59	0.784	0.0176	0.0173	0.0179
*Rep*	162	77	0.952	0.0219	0.0215	0.0223
*Trap*	162	29	0.692	0.0157	0.0154	0.016
*Ren*	162	45	0.804	0.0253	0.025	0.0256
*AC4*	162	25	0.771	0.0178	0.0173	0.0183
*MP*	52	22	0.917	0.0337	0.0321	0.0353
*NSP*	52	25	0.920	0.0407	0.0389	0.0425
**SiYLCV**						
DNA-A	77	59	0.995	0.0135	0.0131	0.0139
DNA-B	43	35	0.990	0.0216	0.0205	0.0227
*CP*	77	26	0.925	0.0198	0.0192	0.0204
*Rep*	77	37	0.963	0.0074	0.0072	0.0076
*Trap*	77	24	0.883	0.0068	0.0067	0.0069
*Ren*	77	27	0.918	0.0117	0.0114	0.012
*AC4*	77	20	0.822	0.0037	0.0036	0.0038
*MP*	43	25	0.971	0.0336	0.0316	0.0356
*NSP*	43	23	0.966	0.0111	0.0107	0.0115
**SimMV**						
DNA-A	17	16	0.993	0.0432	0.0398	0.0466
DNA-B	9	7	0.994	0.0563	0.0482	0.0644
*CP*	17	11	0.956	0.0267	0.0237	0.0297
*Rep*	17	13	0.963	0.0574	0.052	0.0628
*Trap*	17	10	0.926	0.0174	0.0161	0.0187
*Ren*	17	10	0.926	0.0185	0.0174	0.0196
*AC4*	17	10	0.926	0.071	0.0624	0.0796
*MP*	9	6	0.889	0.0342	0.0296	0.0388
*NSP*	9	5	0.833	0.0544	0.0467	0.0621

Although all viruses were sampled from the same host species, large differences in nt diversity were observed among them. To assess whether specific mechanisms were favouring certain types of mutations in the viral genomes, we estimated mutational bias based on base frequencies. We detected multiple significant biases, with a consistent trend across the three species and both genomic components. Among these, C→T and G→A substitutions showed the strongest deviations according to the χ² test (Table 2). These patterns are widely reported in begomoviruses [30, 31, 79] and can be largely explained by cytosine deamination to uracil (read as thymine during replication), adenine deamination to hypoxanthine (which pairs as guanine) and guanine deamination to xanthine (which pairs as thymine, ultimately leading to G→A transitions) [80]. Interestingly, the DNA-B of OxYVV exhibited a significant excess of G→T substitutions, a mutation type commonly linked to oxidative guanine damage. This bias is consistent with the nature of ssDNA genomes, in which exposed bases are more prone to guanine oxidation, generating 8-oxoG that can mispair with adenine during replication [79, 81, 82].

**Table 2.** Mutational biases inferred from the maximum likelihood phylogeny of OxYVV, SiYLCV and SimMV for both DNA-A and DNA-B components, evaluated using χ² tests of significance. Each cell shows, in order, the number of observed substitutions, the expected number of substitutions (in parentheses), the χ² statistic and the corresponding *P*-value

**OxYVV- DNA-A**				
Resulting base				
Original base	A	T	C	G
A	–	35 (18.5), 14.716, 0.00012	21 (13.2), 4.533, 0.033	71.5 (38.0), 29.533, 5.5e^−08^
T	39 (18.5), 22.716, 1.9e^−06^	–	121.5 (71.5), 34.965, 3.4e^−09^	36 (32.0), 0.500, 0.48
C	32 (13.2), 26.533, 2.6e^−07^	164.5 (71.5), 120.965, 3.9e^−28^	–	40 (19.5), 21.551, 3.4e^−06^
G	80.5 (38.0), 47.533, 5.4e^−12^	92 (32.0), 112.500, 2.8e^−26^	38 (19.5), 17.551, 2.8e^−05^	–
**OxYVV- DNA-B**				
Resulting base				
Original base	A	T	C	G
A	–	31 (15.5), 15.500, 8.3e^−05^	16 (7.8), 8.782, 0.003	54 (27.2), 26.259, 3e^−07^
T	31 (15.5), 15.500, 8.3e^−05^	–	65 (37.0), 21.189, 4.2e^−06^	43 (22.8), 18.025, 2.2e^−05^
C	15 (7.8), 6.782, 0.0092	83 (37.0), 57.189, 4e^−14^	–	18 (8.8), 9.779, 0.0018
G	55 (27.2), 28.259, 1.1e^−07^	48 (22.8), 28.025, 1.2e^−07^	17 (8.8), 7.779, 0.0053	–
**SiYLCV-DNA-A**				
Resulting base				
Original base	A	T	C	G
A	–	7 (5.0), 0.800, 0.37	6 (3.5), 1.786, 0.18	25 (14.2), 8.110, 0.0044
T	13 (5.0), 12.800, 0.00035	–	37.5 (27.8), 3.426, 0.064	23 (12.2), 9.434, 0.0021
C	8 (3.5), 5.786, 0.016	73.5 (27.8), 75.426, 3.8e^−18^	–	8 (4.0), 4.000, 0.046
G	32 (14.2), 22.110, 2.6e^−06^	26 (12.2), 15.434, 8.5e^−05^	8 (4.0), 4.000, 0.046	–
**SiYLCV-DNA-B**				
Resulting base				
Original base	A	T	C	G
A	–	20 (10.8), 7.959, 0.0048	12 (6.8), 4.083, 0.043	30 (16.0), 12.250, 0.00047
T	23 (10.8), 13.959, 0.00019	–	50.5 (29.0), 15.940, 6.5e^−05^	20 (12.2), 4.903, 0.027
C	15 (6.8), 10.083, 0.0015	65.5 (29.0), 45.940, 1.2e^−11^	–	24 (9.5), 22.132, 2.5e^−06^
G	34 (16.0), 20.250, 6.8e^−06^	29 (12.2), 22.903, 1.7e^−06^	14 (9.5), 2.132, 0.14	–
**SimMV- DNA-A**				
Resulting base				
Original base	A	T	C	G
A	–	25.5 (11.2), 18.050 2.2e^−05^	18 (7.8), 13.556, 0.00023	36.5 (22.8), 8.310, 0.039
T	19.5 (11.2), 6.050, 0.014	–	82 (42.2), 37.398, 9.6e^−10^	37.5 (19.2), 17.302, 3.2e^−05^
C	13 (7.8), 3.556, 0.059	87 (42.2), 47.398, 5.8e^−12^	–	17 (8.0), 10.125, 0.0015
G	54.5 (22.8), 44.310, 2.8e^−11^	39.5 (19.2), 21.302, 3.9e^−06^	15 (8.0), 6.125, 0.013	–
**SimMV- DNA-B**				
Resulting base				
Original base	A	T	C	G
A	–	41 (18.8), 26.403, 2.8e^−07^	20 (10.0), 10.000, 0.0016	48 (24.2), 23.260, 1.4e^−06^
T	34 (18.8), 12.403, 0.00043	–	70 (36.0), 32.111, 1.5e^−08^	39 (21.0), 15.429, 8.6e^−05^
C	20 (10.0), 10.000, 0.0016	74 (36.0), 40.111, 2.4e^−10^	–	30 (12.2), 25.719, 3.9e^−07^
G	49 (24.2), 25.260, 5e^−07^	45 (21.0), 27.429, 1.6e^−07^	19 (12.2), 3.719, 0.054	–

### Recombination analysis

Since the begomoviruses sampled in this study are part of the same community (with some cases of mixed infections; Table S1), recombination was analysed in an inter-specific data set including sequences from the five viruses. Recombination events involving the DNA-A of at least one individual from each sampled species were identified (Table 3). Only one recombination event was detected for OxYVV, in an isolate (BR_VIC1006_1) of the S2 strain. The event involves a major parent of the same strain and an unknown minor parent. Interestingly, all 77 SiYLCV sequences were identified as recombinants, in an event involving OxYVV as a minor parent and an unknown major parent. Three recombination events were detected for SimMV, one which was strictly intraspecific and the other two involving minor parents of the same species and unknown major parents. Two events were detected for SiMV, the first involving OxYVV and SiYLCV and the second involving SiYLCV and SimMV. We also detected evidence of recombination in MaYVV involving OxYVV as the main parent. Most of the recombination breakpoints were located between the intergenic region and the *Rep* gene, the one exception where the breakpoints were located in the *CP* gene (Table 3).

**Table 3.** Summary of recombination events detected in the begomovirus community infecting *S. acuta* in Viçosa, MG. The analysis was performed using complete DNA-A and DNA-B sequences

Event #	# seq	Breakpoints		Recombinant sequence	Parent		Detection method						
		End	Begin		Minor	Major	RDP	Geneconv	Bootscan	Maxchi	Chimaera	SiSscan	3Seq
**DNA-A**													
1	1	303*	31	OxYVV BR_Vic_1006_1	OxYVV BR_Vic_917_2	Unknown	1.316e^−18^	1.799e^−18^	1.932e^−13^	9.167e^−11^	4.331e^−12^	9.380e^−09^	1.027e^−28^
2	5	91	2245	SiMV BR_Vic_924_1	SiYLCV BR_Vic_907_8	OxYVV BR_Vic_917_2	1.961e^−20^	2.684e^−23^	1.532e^15^	4.167e^−17^	1.486e^−13^	1.778e^−27^	1.573e^−10^
3	1	1893	2596	MaYVV BR_Vic_911_4	OxYVV BR_Vic_1012_1	Unknown	5.355e^−9^	ns	8.853e^−11^	1.118e^−18^	4.841e^−8^	1.101e^−22^	8.163e^−22^
4	3	2227	133	SimMV BR_Vic_837_5	SimMV BR_Vic_736_2	Unknown	2.818e^−7^	3.445e^−3^	6.757e^−7^	2.420e^−14^	2.863e^−5^	5.337e^−8^	3.629e^−10^
5	77	194	2042	SiYLCV BR_Vic_907_8	OxYVV BR_Vic_1020_2	Unknown	5.747e^−9^	2.858e^−5^	3.392e^−4^	3.132e^−10^	1381e^−11^	3.536e^−24^	ns
6	1	1832	31	SimMV BR_Vic_913_4	SimMV BR_Vic_608_1	SiMMV BR_Vic_810_2	8.396e^−6^	ns	9.318e^−5^	2.318e^−13^	1.902e^−8^	ns	3.436e^−3^
7	6	31	2241	SimMV BR_Vic_810_2	SimMV BR_Vic_618_2	Unknown	3.321e^−6^	8.206e^−6^	1.278e^−5^	4.944e^−7^	1.482e^−5^	8.290e^−19^	3.172e^−4^
8	4	893	673	SiMV BR_Vic_924_1	SiYLCV BR_Vic626_1	SimMV BR_Vic_618_2	8.641e^−4^	4.256e^−5^	1.369e^−3^	ns	2.582e^−2^	5.368e^−3^	2.871e^−3^
**DNA-B**													
1	35	1884	1546	SiYLCV BR_Vic_411_3	Unknown	SiYLCV BR_Vic_1009_1	1.837e^−21^	4.342e^−19^	2.637e^−18^	1.982e^−7^	9.626e^−8^	1.287e^−7^	5.387e^−12^
2	1	2616	2514	SiMV BR_Vic_0912_10	OxYVV BR_Vic_1014_06	SiMV BR_Vic_0632_27	4.75e^−12^	1.02e^−13^	3.22e^−3^	6.48e^−07^	6.57e^−7^	2.14e^−10^	1.37e^−12^
3	1	1770	1436	BR_Vic_0525_1*	SiYLCV BR_Vic_1009_1	Unknown	4.18e^−6^	8.37e^−10^	1.81e^−7^	6.41e^−05^	2.74e^−3^	1.95e^−09^	ns
4	2	2598	2486	SiMV BR_Vic_0632_27	Unknown	OxYVV BR_Vic_0816_3	2.36e^−9^	2.71e^−7^	1.15e^−6^	1.24e-2	4.79e^−5^	4.27e^−11^	1.97e^−7^
5	1	2624	2503	SimMV BR_Vic_0726_06	SimMV BR_Vic_0607_07	SimMV BR_Vic_0712_4	1.95e^−4^	5.32e^−8^	6.18e^−7^	1.35e-3	3.15e^−3^	1.62e-8	ns
6	5	2544	1145	SiYLCV BR_Vic_1009_1	SiYLCV BR_Vic_0622_24	SiYLCV BR_Vic_0905_2	1.82e^−3^	2.49e^−3^	ns	2.30e-7	1.64e^−3^	4.29e^−10^	3.27e^−7^
7	44	2485	2608	SiYLCV BR_Vic_0804_2	OxYVV BR_Vic_0816_3	Unknown	ns	ns	ns	2.70e-9	1.77e^−5^	2.78e^−38^	9.95e^−6^

* Numbering starts at the first nt after the cleavage site at the origin of replication and increases clockwise

For the DNA-B component, no significant recombination events were identified in the OxYVV population (Table 3). Conversely, three recombination events were detected for SiYLCV. Event 7 was detected in all SiYLCV sequences analysed and involved OxYVV and an unknown major parent. The second event was observed in 35 SiYLCV sequences, with the major parent unknown and the minor parent being SiYLCV. The third event was an intraspecific recombination event detected in five SiYLCV sequences. A single SimMV DNA-B sequence showed evidence of intraspecific recombination. The breakpoints detected in the DNA-B varied in their location (Table 3). For four of the seven events (events 2, 4, 5 and 7), the breakpoints were found in the larger intergenic region (LIR). The breakpoints of events 1 and 3 are located within the *MP* gene, while those of event 5 spanned part of the *NSP* gene up to the LIR.

The evolutionary history and the impact of recombination were captured by reconstructing phylogenetic networks. These networks, unlike phylogenetic trees, allow us to visualize the occurrence of lateral transfers of genomic regions between individuals. The DNA-A network formed five large clusters corresponding to each of the five viruses (Fig. 3a). The OxYVV cluster showed a clear division into six dense clusters with little cross-linking between them. One of these cross-links connects the BR_Vic_1006_1 isolate (OxYVV-S2 strain) to the other individuals affiliated with this strain, indicating a pattern of genetic exchange. SiYLCV formed a group separated by a long branch with rare reticulations. Some reticulations connected the SiMV and OxYVV-S2 groups, indicating patterns of recombination between these viruses. Some edges are shared within the SimMV cluster. Also, exchange patterns were found between SimMV and MaYVV.

**Fig. 3.**
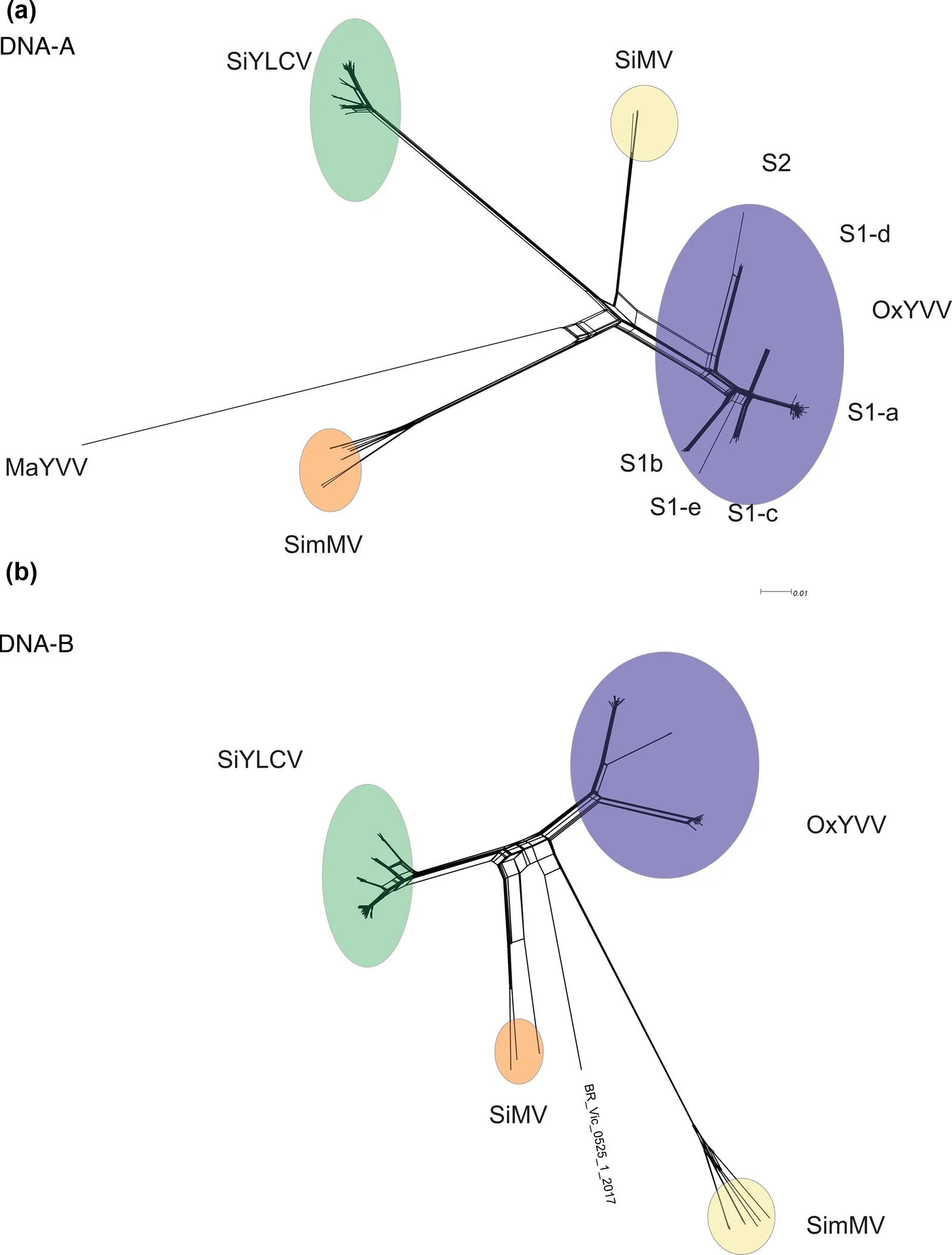
Phylogenetic evidence of recombination in the DNA-A (**a**) and DNA-B (**b**) sequences of the begomovirus community infecting *S. acuta* in Viçosa, MG. Neighbour-Net network analysis was performed using SplitsTree4. The formation of a reticular network rather than a single bifurcated tree suggests recombination.

The DNA-B network also presented cross-linking patterns. Five clusters were formed corresponding to each of the viruses (including the one for which the species was not identified). Edges connecting SiMV individuals were observed. Some side branches also connected SimMV and the unidentified virus, indicating possible recombination events (Fig. 3b).

We inferred the relative contribution of recombination and mutation to the diversification of the *S. acuta* begomovirus community by calculating the ratio between these two rates (ρ/θ). For both components and most data sets, the ρ/θ ratio was <1 (Table 4), indicating a greater probability of polymorphisms being the result of mutation rather than recombination. The only case where there was a greater relative contribution of recombination was for SiYLCV (Table 4). Even though most of the ratios were <1, they can be considered high, revealing the important role of recombination as a driver of diversification.

**Table 4.** Population-scaled rates of recombination and mutation of the begomoviruses infecting *S. acuta* in Viçosa, MG

Species/segment	S	ρ	θ_W_	ρ/θ_W_
OxYVV DNA-A	470	45.67	90.96	0.502
OxYVV DNA-B	393	29.04	86.97	0.333
SiYLCV DNA-A	202	69.81	41.33	1.689
SiYLCV DNA-B	269	40.50	61.83	0.655
SimMV DNA-A	303	30.61	89.63	0.341

S, number of segregating sites; ρ, population-scaled recombination rate; θw, Watterson’s estimator of population-scaled mutation rate (θ); ρ/θ, recombination/mutation ratio.

### Evidence of temporal signal in the OxYVV and SiYCV populations

Recombination events can distort branch lengths and inflate substitution rate estimates [25, 83]. Only one OxYVV sequence showed evidence of a recombination event, and this sequence was not used for substitution rate estimation. SiYLCV, on the other hand, showed evidence of recombination in all 77 sequences analysed; however, this corresponds to a single event shared by all of them, suggesting that the most parsimonious scenario is that of a common ancestor that already carried this recombination event. Taking this into account, the SiYLCV sequences analysed here diversified through the accumulation of substitutions, since no additional secondary recombination events were observed in the population; therefore, this does not violate the underlying assumption. We employed BETS to infer evidence of a temporal signal in the data sets. For both OxYVV and SiYLCV, the higher marginal likelihood values were observed under the heterochronous model. A Bayes factor greater than 5 indicates that providing sampling dates allowed for successful calibration of the molecular clock, representing strong evidence of a temporal signal (Table 5). The BETS analysis was also conducted on the *CP* and *Rep* genes of both OxYVV and SiYLCV, with (log) Bayes factor values supporting strong evidence of a temporal signal (Table 5).

**Table 5.** Detection of a temporal signal in the begomovirus populations infecting *S. acuta* in Viçosa, MG, using BETS. The log marginal likelihood was estimated for both isochronous data (without sampling dates) and heterochronous data (with sampling dates), under both strict and relaxed molecular clock models. The log Bayes factor was calculated as the difference between the log marginal likelihoods of the two competing models. Analyses were conducted for the DNA-A, *Rep* and *CP* genes of OxYVV and SiYLCV. Log Bayes factors greater than five indicate strong statistical support favouring heterochronous models over isochronous ones

	Strict clock	Relaxed clock
OxYVV DNA-A		
Without sampling dates	−9717.87	−9653.157146
With sampling dates	−9629.50	−9596.735873
Log Bayes factor	88.37	56.42127296
OxYVV *Rep*		
Without sampling dates	−3846.46	−3890.83
With sampling dates	−3777.28	−3780.82
Log Bayes factor	69.18	110.01
OxYVV *CP*		
Without sampling dates	−2507.33	−2509.76
With sampling dates	−2446.65	−2440.98
log Bayes factor	60.68	68.78
SiYLCV DNA-A		
Without sampling dates	−6155.14	−6148.02
With sampling dates	−6107.14	−6101.66
Log Bayes factor	47.99	46.36
SiYLCV *Rep*		
Without sampling dates	−2252.04	−2266.35
With sampling dates	−2206.64	−2209.55
Log Bayes factor	45.39	56.79
SiYLCV *CP*		
Without sampling dates	−1807.04	−1791.22
With sampling dates	−1754.16	−1747.22
Log Bayes factor	52.87	44.01

### Substitution rates of OxYVV and SiYLCV

We estimated the number of substitutions per site per year for the DNA-A, *Rep* and *CP* genes of both viruses (Fig. 4). The mean substitution rates were in the same order of magnitude across the genomic components of both OxYVV and SiYLCV. For OxYVV, the mean substitution rate was 4.81×10^−4^ for the DNA-A, 8.26×10^−4^ for the *CP* gene and 2.54×10⁻^4^ for the *Rep* gene (Fig. 4). For SiYLCV, the rate was 4.33×10^−4^ for the DNA-A, 3.17×10⁻^4^ for *Rep* and 1.21×10^−3^ for the *CP* gene, the higher value observed (Fig. 4).

**Fig. 4.**
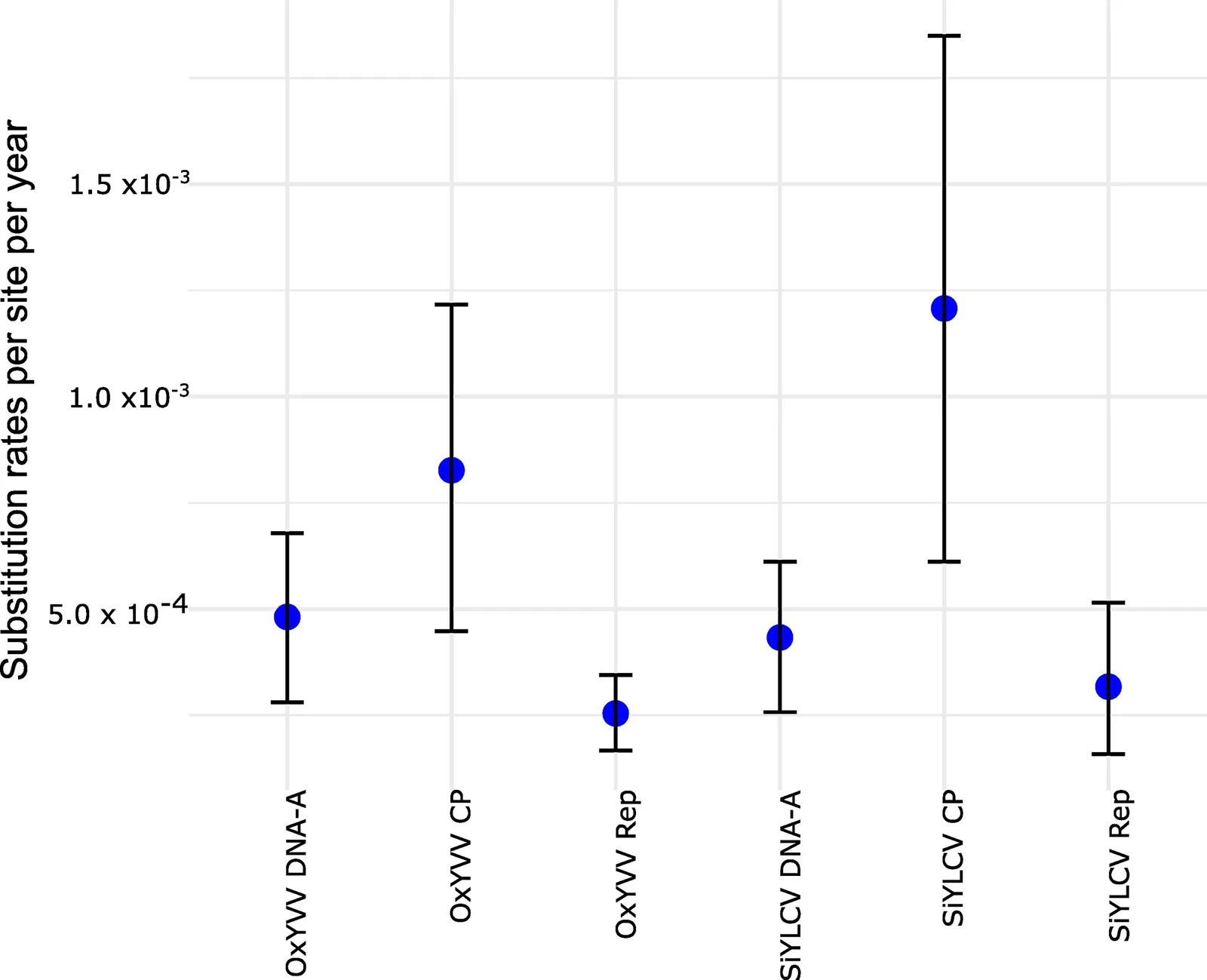
Substitution rates (nts per site per year) for OxYVV and SiYLCV. Estimated mean substitution rates and corresponding 95% HPD intervals are shown for the complete DNA-A, the coat protein (*CP*) gene and the replication-associated protein (*Rep*) gene of OxYVV and SiYLCV. Estimates were derived using a Bayesian molecular clock framework.

The substitution rates of the complete genomes and the *Rep* genes of the two viruses did not differ statistically (Fig. 4). However, for both viruses, the substitution rates of the *CP* gene were significantly higher compared to those of the *Rep* gene. The estimated substitution rates align with those estimated for other begomoviruses [30, 31, 84] and are comparable to those observed for ssRNA viruses.

### Time of the most recent common ancestor

The estimated TMRCA varied across the genomic components of both viruses. For OxYVV, the TMRCA of the complete DNA-A was estimated to be ~1931, with a 95% HPD interval between 1858 and 1991 (Fig. 5a). The OxYVV *CP* gene had a more recent TMRCA of 1985, with an HPD interval of 1937–2005 (Fig. S7A). The OxYVV *Rep* gene showed the oldest TMRCA, estimated at 1825, with an HPD interval of 1730–1888 (Fig. S8A). For SiYLCV, the TMRCA of the complete DNA-A component was estimated at 1991, with a 95% HPD interval ranging from 1965 to 2005 (Fig. 5b). The SiYLCV *CP* gene had the most recent TMRCA, estimated at 2008, with an HPD interval of 1990–2011 (Fig. S7B). The SiYLCV *Rep* gene TMRCA was estimated to be 1995, with a 95% HPD interval between 1975 and 2007 (Fig. S8B).

**Fig. 5.**
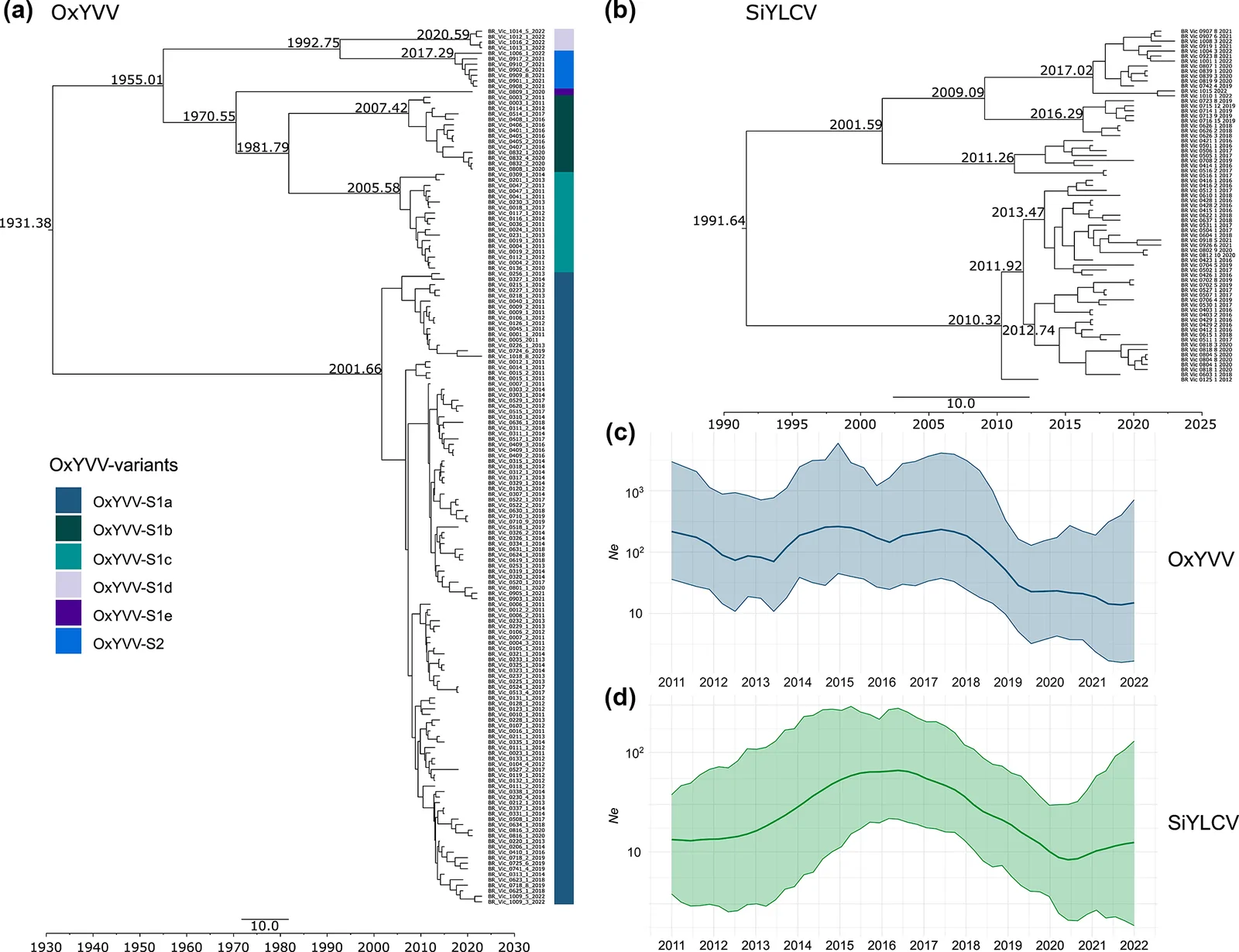
Evolutionary and demographic dynamics of OxYVV and SiYLCV. (**a)** MCC phylogenetic tree for OxYVV. The numbers on the branches indicate the TMRCA. The identified OxYVV variants (S1a–S1e and S2) are shown in distinct colours in the side bar.** (b)** MCC tree for SiYLCV. (**c, d)** Demographic history under a coalescent framework (effective population size, Ne) inferred using the Bayesian Skygrid model for OxYVV (**c**) and SiYLCV (**d**). The lines represent the median estimates, and the shaded area corresponds to the 95% HPD intervals.

### Demographic history in a coalescent framework

We hypothesized that the shifts in the composition of the viral community could have been caused by an event that led to a reduction in the effective population size of OxYVV, the prevalent species in the first 4 years. This may have created an opportunity for SiYLCV to outcompete OxYVV. To test this hypothesis, we reconstructed the demographic trajectory of OxYVV and SiYLCV populations through time using the Bayesian Skygrid Coalescent Model [71].

The OxYVV population exhibited expansion and contraction dynamics. The first expansion occurred ~2010, reaching a peak in 2016, followed by a decline that extended from mid-2017 to 2022 (Fig. 5c). However, there is a degree of uncertainty in the estimation of the effective population size (Ne), which limits our ability to draw conclusions about possible bottlenecks that could explain the changes observed in 2016. We also measured changes in Ne for the OxYVV *Rep* and OxYVV *CP* genes. The OxYVV *Rep* gene showed a pattern similar to that observed for the complete genome (Fig. S8C). In contrast, the OxYVV *CP* gene exhibited an almost constant Ne over time (Fig. S7C).

SiYLCV showed an increase in effective population size from 2012 to 2016, followed by a reduction after 2016 (Fig. 5d). The pattern for the SiYLCV *Rep* gene resembled that of the DNA-A (Suppl. Figure S8C), whereas the *CP* gene showed a nearly constant Ne over time (Fig. S7C). The population dynamics captured by the Skygrid model reflect the variation in the genetic diversity of OxYVV and SiYLCV populations over time and are consistent with the hypothesis that a decline in the OxYVV population allowed for the increase in SiYLCV.

### Predominance of purifying selection in the populations

Neutrality tests were performed to investigate the role of selection in different coding regions. The tests were performed for the OxYVV and SiYLCV data sets but not for SimMV, due to the small number of sequences in this data set. We obtained negative and significant values for Tajima’s D, Fu and Li D* and Fu and Li F* tests in most genes of OxYVV and SiYLCV (Table S4), indicating an abundance of rare alleles in the populations and suggesting that these genes underwent purifying selection or a recent expansion, as observed in the previous section with the Skygrid analysis.

To identify the type of selection that is acting at the aa level in each region of the genome, we estimated ω (dN/dS) using the SLAC method. Most genes showed values of ω<1, indicating that purifying selection is acting on them (Table S4). The only exception was the *AC4* gene, which is under diversifying selection for both OxYVV and SiYLCV (Table S4). The OxYVV *CP*, *Rep*, *MP* and *NSP* genes are under strong negative selection, indicated by very low values of ω. On the other hand, the OxYVV *TrAP* and *REn* genes seem to experience more relaxed negative selection (Table S4). The SiYLCV *CP*, *MP* and *NSP* genes are also subject to strong purifying selection (Table S4).

In addition, we analysed the distribution of substitutions by codon position across the coding regions and mapped them for OxYVV, SiYLCV and SimMV (Table S5). The three viruses showed very similar patterns regarding the distribution of substitutions by codon position. For example, the *CP*, *Rep*, *MP* and *NSP* genes exhibited a higher percentage of substitutions at the third codon position, which reflects a higher proportion of synonymous substitutions, consistent with the ω estimates. In contrast, the *TrAP* and *REn* genes presented similar levels of substitutions at the first and third codon positions, while *AC4* showed a higher percentage of non-synonymous substitutions at the second codon position, resulting in more aa changes. These patterns are consistent with what would be expected under diversifying selection.

Using SLAC, we identified several aa sites under negative selection and none under positive selection in the data sets. Using FUBAR, we were able to identify a larger number of sites under selection, including those detected by SLAC and several others also under negative selection, in addition to detecting some sites under positive selection (Table S4). We also found several sites under positive selection using MEME. In general, the *CP*, *Rep* and *MP* genes exhibited a higher number of sites under selection, with OxYVV presenting the largest number of sites (Table S4). Together, these results indicate that the two viruses experience slightly different selection regimes, evidenced by the number of sites detected in each of the data sets.

## Discussion

Longitudinal, hypothesis-driven sampling of viral populations in natural landscapes can provide valuable insights into the ecological interactions and evolutionary trajectories that shape viral community diversity. Here, we monitored begomoviruses infecting the wild host *S. acuta* over more than a decade within a small area in a natural environment and investigated the contributions of multiple evolutionary mechanisms underlying community dynamics. Such approaches offer opportunities to uncover intrinsic evolutionary processes operating in natural populations and allow us to draw parallels with agricultural systems, enabling assessment of how anthropogenic activity may inadvertently alter viral evolutionary trajectories. Importantly, temporal dynamics assessed through systematic, longitudinal sampling have the potential to mitigate potential artefacts associated with uneven temporal and spatial sampling, particularly in fragmented populations exhibiting strong genetic structure [39].

The role of non-cultivated plants, especially those from the Malvaceae family, as reservoirs of begomovirus diversity has been demonstrated by several studies [11, 85–90]. We show that even in a small area, *S. acuta* can harbour complex communities composed of multiple species, variants and co-occurring strains. Thus, *S. acuta* is a ‘mixing vessel’ host, which are important players from an evolutionary and ecological perspective, serving as reservoirs and sources of viral genetic diversity. Furthermore, our results demonstrate that the distribution of species diversity over time is not static, with major changes in the dominance of species and variants over the years. The most intriguing observation from our study is the increase in species diversity in the area, from a single virus in 2011 to five viruses in 2021, with a corresponding doubling of the KHILL value during this period. The number of species remained constant from 2011 until 2014, with a sudden change in 2016, followed by a steady increase until 2021. This reveals the need for continuous surveillance in this type of community, as this has the potential to provide insights into how the composition of viruses circulating in wild plants may be associated with outbreaks observed in the field.

A hypothesis to explain the changes in species composition observed in 2016 would be the occurrence of a random event which resulted in a drastic reduction in the effective size of the OxYVV population. This genetic bottleneck could have altered the adaptive landscape, creating an opportunity for SiYLCV to become established in the area [91, 92]. However, the variations in effective population size over time do not provide any evidence of a genetic bottleneck that would have significantly reduced the diversity of OxYVV. An alternative explanation would be a fitness increase of SiYLCV following a recombination event (all isolates of SiYLCV obtained in our work are recombinants). Frequency changes alone are insufficient to demonstrate increased fitness; we are currently constructing infectious clones to experimentally assess the relative fitness of SiYLCV and OxYVV. Other local emergence events were observed, including SimMV, SiMV and MaYVV. These viruses could have been introduced through lateral transmission from other sources. It is also possible that they were present in the area from the beginning but at extremely low frequencies, such that the probability of them being amplified by RCA and then cloned would be negligible, as shown by Quadros *et al*. [93] for SimMV in tomato and tomato severe rugose virus in *Sida micrantha*. Their detection in the late years of our sampling could be the result of the above-mentioned bottleneck or fitness increase.

Although it would be tempting to speculate on all possible mechanisms that could explain the changes observed in the viral community, our study was not actually designed to address these questions. The observed turnover and the emergence of additional species in the site were outcomes of long-term longitudinal monitoring; these observations open avenues for future research. Our results highlight the need for further longitudinal studies that incorporate additional players of the ecological network, such as multiple plant host species, insect vectors and even the vector’s microbiome. The breadth of the host range of begomoviruses can vary, and some species exhibit broader host ranges than others, implying the existence of multiple reservoirs [17, 94, 95]. As has been demonstrated, different species of the *Bemisia tabaci* complex display distinct foraging behaviour and transmission efficiencies, and the vector microbiome may influence virus transmission efficiency [96, 97].

Various emergence events of new viruses in the area over time were observed, some of them occurring at low frequency, such as SiMV, MaYVV and SimMV. Although SimMV persisted over time, its prevalence in the area remained low compared to OxYVV and SiYLCV. In contrast, the emergence of SiYLCV was particularly notable, as it was associated with the displacement of the previously dominant local species (OxYVV). This pattern highlights the potential of this virus for ecological niche expansion and possibly spillover. Although our study focused on a single host species and did not include vector sampling, it is reasonable to speculate that, under favourable conditions, such as the presence of a competent vector and susceptible hosts, there is a risk of spillover into new host systems. Similar events involving host shifts from wild to cultivated hosts, followed by displacement of previously dominant viral species, have been documented in begomoviruses. For example, the emergence of the recombinant East African cassava mosaic virus-Uganda (EACMV-UG) was associated with the displacement of other cassava mosaic begomoviruses [98, 99]. Comparable patterns have also been reported for tomato-infecting begomoviruses of the TYLCV complex, where invasive or recombinant variants have reshaped local viral populations [100, 101]. The SiYLCV population analysed here appears to have emerged through recombination, which may have contributed to its successful establishment. Together, these observations reinforce the importance of longitudinal monitoring for understanding the risks of spillover, not only within agricultural systems but also across natural landscapes. Such dynamics may have broader implications, including potential impacts on conservation areas, where the introduction of novel viruses into native plant communities could affect ecological balance and promote further viral diversification.

Interestingly, despite infecting the same host (*S. acuta*) in the same area, the viruses detected in our study exhibited distinct levels of genetic variability: SimMV exhibited the highest genetic variability, followed by OxYVV, whereas SiYLCV exhibited the lowest variability. These patterns were consistent across both genomic components, DNA-A and DNA-B. Although a logical expectation would be similar levels of variability, given that these viruses rely on host polymerases for replication, this represents an overly simplistic assumption that has long been challenged by several studies demonstrating the independence between begomovirus genetic variability and host identity [17, 94, 95]. Although the accumulation of variability does depend on the fidelity of the host DNA polymerases, this is not the only factor involved. Rather, the accumulation of genetic variability reflects the combined action of multiple processes operating simultaneously, including infection dynamics, genome architecture, gene function and their interactions with host and vector, as well as selection regimes and demographic processes.

The DNA-B components of all three major viruses detected in the study area showed higher variability than their cognate DNA-A components, as previously reported for begomoviruses infecting both non-cultivated and cultivated hosts [77, 78]. There is direct and indirect evidence of differential accumulation between the two components, favouring the DNA-B [102, 103]. Higher replication rates increase the probability of incorrect base incorporation [30]. In addition, differential accumulation of genomic components may reduce the strength of transmission bottlenecks for the DNA-B [102, 104]. This framework may also apply to DNA-A components across species that exhibit distinct replication levels. Moreover, large regions of the DNA-A contain overlapping genes, which can impose strong selective constraints against mutations in these regions [103, 105, 106]. Another factor to consider is divergence time: species that emerged more recently may not yet have had sufficient time to accumulate variability in comparison to a species that has been diversifying for a long time. Our reconstructions of the TMRCA provide clues about this. While the inferred TMRCA for the more variable OxYVV population dates to 1931, that of the less variable SiYLCV was estimated at 1991. An observation that further reinforces this hypothesis concerns the levels of genetic variability observed for the *CP* and *Rep* genes, which may at first seem counterintuitive. The *Rep* gene shows higher variability than the *CP* gene, yet *CP* presents higher substitution rates. However, when we consider the TMRCA for each gene, this pattern becomes more coherent. The *Rep* genes of OxYVV and SiYLCV appear to have diverged earlier than the *CP* genes, allowing more time for the accumulation of standing variation (Figs S7 and S8). The distinct evolutionary patterns observed for these genes may reflect partially independent evolutionary histories, possibly associated with recombination-driven speciation, although this was not formally evaluated here. The relationship between divergence time and the degree of observed variability requires further investigation in begomoviruses.

The role of recombination in begomovirus evolution is well recognized and is frequently associated with the successful emergence of begomoviruses in crops [98, 100, 107–109]. However, it has been shown that the relative contribution of mutations as a variability-generating mechanism is a more important role than that of recombination [25, 27, 83]. Interestingly, this did not apply to SiYLCV, the first begomovirus that deviates from previous observations. Indeed, every SiYLCV genomic component cloned in our study is a recombinant, and many of them were multiple recombinants. Moreover, OxYVV was inferred as one of the parental sequences in most recombination events. SiYLCV emerged in the area in 2016 and quickly became the predominant virus (although in later years its relative incidence decreased). Thus, it is not unreasonable to propose that one or more of the recombination events led to a fitness increase of SiYLCV and thus to its emergence and predominance in the area. Our current studies are focused on comparing the fitness of OxYVV and SiYLCV isolates using controlled inoculation experiments with infectious clones and with the insect vector.

We observed variation in the number of recombination events detected among the three predominant species in the area: SimMV showed the highest number of events, whereas OxYVV presented the lowest number of recombination events, with only a single recombinant sequence. Several factors may be associated with the range of different species that can infect a given host, which would create opportunities for co-infections, a condition favourable for the occurrence of these events [110–112]. However, co-infection events were less frequent than single-infection events, which may be due to the strategy employed to generate our data, which involved an amplification step that could have favoured preferential amplification of more abundant viruses in the sample. The use of strategies such as high-throughput sequencing without an amplification step could capture these nuances. On the other hand, the strategy we employed, involving genome cloning, allows the reconstruction of real haplotypes and mitigates potential genome assembly artefacts that could inadvertently produce chimeric haplotypes, resulting in the detection of false recombination events.

Overall, we detected a higher number of unique recombination events in DNA-A than in DNA-B: while we detected eight events for the former, we detected only seven for the latter. Interestingly, the number of recombination events reported was not a function of dataset size; for example, OxYVV, for which we have the largest number of sequences, was the species for which we detected the fewest recombination events for both components. Conversely, the SimMV DNA-A (a species for which we have only 17 sequences) presented the highest number of unique recombination events. Although one of our own previous studies [78] reported higher recombination frequencies for the DNA-B compared to the DNA-A, our study differs in terms of scale, landscape type and the number of hosts sampled. While our study is restricted to a single host in a single location in a natural environment, Xavier *et al*. [78] focused on larger geographic scales and multiple hosts, which increases the probability of sampling recombinant sequences.

Bayesian inference indicated that substitution rates estimated for both OxYVV and SiYLCV are of the same order of magnitude as those reported in previous studies, namely between 10⁻³ and 10⁻⁴ substitutions per site per year [30, 31]. This reinforces the consistency of high evolutionary rates in begomoviruses and provides evidence that earlier estimates are robust even though previous datasets were subject to several limitations. Moreover, our study shows that begomoviruses evolving in natural environments diversify at rates comparable to those observed in agricultural systems, providing additional support for the idea that rapid evolution is an intrinsic feature of these viruses.

Some aspects were not fully covered in our study and this prevents us from drawing concrete conclusions about the mechanisms underlying the changes in community composition. One of them is the restriction of our sampling to a single host species and a single location, which prevents us from determining the source of the emerging viral species. These viruses that were detected could have been circulating in other hosts in the same area or may have come from nearby areas. Another limitation was the absence of vector sampling, which could provide evidence allowing us to correlate changes in the viral composition of the landscape with changes in the genetic composition of the vector or even its microbiome. Similarly, we did not explore the association of climatic variables, such as temperature, humidity and rainfall over the sampling period. These variables are known to affect whitefly population fluctuations and could therefore provide relevant insights into the temporal dynamics of the species composing the community. Finally, the sequencing approach we used, based on amplification and cloning, may have limited the detection of the true composition of the viral community, since some of the viruses that emerged later could have already been infecting the sampled plants but at levels below the detection limit. Although these aspects were not addressed in this study, they highlight important gaps in our understanding of the ecological network involving begomoviruses in natural environments. At the same time, they open an avenue to explore the ecological and evolutionary dynamics of begomoviruses from a network perspective, integrating multiple hosts, the vector and its microbiome, and how these factors shape changes in community structure.

In conclusion, our results reinforce the role of wild plants as reservoirs of viral biodiversity and reveal that begomoviruses coexist as complex communities composed of distinct species, further subdivided into strains and genetic variants. Community composition was highly dynamic, with changes of dominant types across the landscape and multiple local emergence events. The processes governing these compositional shifts may include the high evolutionary rates of the two most sampled viruses (OxYVV and SiYLCV) and also the high recombination rate of SiYLCV, which may have generated a viral variant with higher fitness. We are currently investigating aspects associated with differential fitness among the viruses comprising this landscape. This work also opens new directions for research on begomovirus community dynamics and highlights the importance of considering additional players in the complex ecological network underlying begomovirus persistence and spread. Our study demonstrates, for the first time, that begomovirus evolutionary rates remain high even in an environment with minimal human intervention.

## Supplementary material

10.1099/jgv.0.002340 Supplementary Material 1.

## Abbreviations

BETS: Bayesian evaluation of temporal signal
FUBAR: Fast Unconstrained Bayesian AppRoximation
HPD: highest posterior density
LIR: larger intergenic region
MaYVV: Macroptilium yellow vein virus
MCC: Maximum Clade Credibility
MCMC: Markov chain Monte Carlo
MEME: Mixed Effects Model of Evolution
OxYVV: Oxalis yellow vein virus
RCA: rolling circle amplification
SimMV: *Sida micrantha* mosaic virus
SiMV: Sida mottle virus
SiYLCV: Sida yellow leaf curl virus
SLAC: Single Likelihood Ancestor Counting
TMRCA: time to the most recent common ancestor
