# Supplementary material for: Into the wild: a long-term study of begomovirus community dynamics in a natural landscape
Source: J Gen Virol. 2026 Oct 12;107(10):002340. doi: 10.1099/jgv.0.002340 (PMC13657429; doi:10.1099/jgv.0.002340)
Supplement: Supplementary file 1 [file jgv-107-02340-s001.pdf]

Supplementary Table S1. *Sida acuta* samples collected in Viçosa, MG, from 2011 to 2022, and the corresponding begomovirus clones obtained from each sample. Samples indicated in bold had a mixed infection.

Sample	Year	Isolate	Genbank acc. number	Comp.	Enzyme	Species- variant
01	2011	BR_Vic_0001_1_2011	PX277586	DNA-A	ClaI	OxYVV-S1a
		BR_Vic_0001_1_2011	PX277748	DNA-B	BamHI	OxYVV
03	2011	BR_Vic_0003_1_2011	PX277587	DNA-A	ClaI	OxYVV-S1b
		BR_Vic_0003_2_2011	PX277588	DNA-A	ClaI	OxYVV-S1b
		BR_Vic_0003_1_2011	PX277769	DNA-B	BamHI	OxYVV
		BR_Vic_0003_2_2011	PX277770	DNA-B	BamHI	OxYVV
		BR_Vic_0003_3_2011	PX277774	DNA-B	BamHI	OxYVV
04	2011	BR_Vic_0004_1_2011	PX277589	DNA-A	ClaI	OxYVV-S1c
		BR_Vic_0004_2_2011	PX277590	DNA-A	EcoRV	OxYVV-S1c
		BR_Vic_0004_3_2011	PX277591	DNA-A	ClaI	OxYVV-S1a
05	2011	BR_Vic_0005_2011	PX277592	DNA-A	ClaI	OxYVV-S1a
		BR_Vic_0005_3_2011	PX277759	DNA-B	BamHI	OxYVV
06	2011	BR_Vic_0006_1_2011	PX277593	DNA-A	ClaI	OxYVV-S1a
		BR_Vic_0006_1_2011	PX277762	DNA-A	EcoRV	OxYVV-S1a
		BR_Vic_0006_2_2011	PX277594	DNA-B	BamHI	OxYVV
07	2011	BR_Vic_0007_1_2011	PX277595	DNA-A	ClaI	OxYVV-S1a
		BR_Vic_0007_2_2011	PX277596	DNA-A	ClaI	OxYVV-S1a
		BR_Vic_0007_1_2011	PX277752	DNA-B	BamHI	OxYVV
		BR_Vic_0007_2_2011	PX277753	DNA-B	BamHI	OxYVV
09	2011	BR_Vic_0009_1_2011	PX277597	DNA-A	ClaI	OxYVV-S1a
		BR_Vic_0009_2_2011	PX277598	DNA-A	EcoRV	OxYVV-S1a
		BR_Vic_0009_1_2011	PX277749	DNA-B	BamHI	OxYVV
10	2011	BR_Vic_0010_1_2011	PX277599	DNA-A	ClaI	OxYVV-S1a
		BR_Vic_0010_1_2011	PX277755	DNA-B	BamHI	OxYVV
12	2011	BR_Vic_0012_1_2011	PX277600	DNA-A	ClaI	OxYVV-S1a
		BR_Vic_0012_2_2011	PX277601	DNA-A	ClaI	OxYVV-S1a
		BR_Vic_0012_1_2011	PX277761	DNA-B	BamHI	OxYVV
13	2011	BR_Vic_0013_1_2011	PX277756	DNA-B	BamHI	OxYVV
		BR_Vic_0013_2_2011	PX277750	DNA-B	BamHI	OxYVV
14	2011	BR_Vic_0014_1_2011	PX277602	DNA-A	ClaI	OxYVV-S1a
		BR_Vic_0014_2_2011	PX277760	DNA-B	BamHI	OxYVV
15	2011	BR_Vic_0015_1_2011	PX277603	DNA-A	ClaI	OxYVV-S1a
		BR_Vic_0015_2_2011	PX277604	DNA-A	ClaI	OxYVV-S1a
		BR_Vic_0015_1_2011	PX277751	DNA-B	BamHI	OxYVV

		BR_Vic_0015_1_2011	PX277751	DNA-B	BamHI	OxYVV
16	2011	BR_Vic_0016_1_2011	PX277605	DNA-A	ClaI	OxYVV-S1a
		BR_Vic_0016_2_2011	PX277786	DNA-B	ClaI	OxYVV-S1a
		BR_Vic_0016_3_2011	PX277776	DNA-B	ClaI	OxYVV-S1a
18	2011	BR_Vic_0018_1_2011	PX277606	DNA-A	EcoRV	OxYVV-S1c
		BR_Vic_0018_1_2011	PX277780	DNA-B	ClaI	OxYVV-S1c
19	2011	BR_Vic_0019_1_2011	PX277607	DNA-A	ClaI	OxYVV-S1c
		BR_Vic_0019_2_2011	PX277608	DNA-A	ClaI	OxYVV-S1c
		BR_Vic_0019_1_2011	PX277782	DNA-B	BamHI	OxYVV-S1c
20	2011	BR_Vic_0020_1_2011	PX277775	DNA-B	ClaI	OxYVV
23	2011	BR_Vic_0023_1_2011	PX277609	DNA-A	ClaI	OxYVV-S1a
		BR_Vic_0023_1_2011	PX277754	DNA-B	BamHI	OxYVV
24	2011	BR_Vic_0024_1_2011	PX277610	DNA-A	ClaI	OxYVV-S1c
27	2011	BR_Vic_0027_1_2011	PX277771	DNA-B	ClaI	OxYVV
32	2011	BR_Vic_0032_2_2011	PX277777	DNA-B	BamHI	OxYVV
36	2011	BR_Vic_0036_1_2011	PX277611	DNA-A	ClaI	OxYVV-S1c
40	2011	BR_Vic_0040_1_2011	PX277612	DNA-A	ClaI	OxYVV-S1a
		BR_Vic_0040_1_2011	PX277793	DNA-B	ClaI	OxYVV-S1a
41	2011	BR_Vic_0041_1_2011	PX277613	DNA-A	ClaI	OxYVV-S1c
		BR_Vic_0041_3_2011	PX277795	DNA-B	ClaI	OxYVV-S1c
42	2011	BR_Vic_0042_1_2011	PX277778	DNA-B	ClaI	OxYVV
		BR_Vic_0042_2_2011	PX277787	DNA-B	ClaI	OxYVV
44	2011	BR_Vic_0044_1_2011	PX277757	DNA-B	BamHI	OxYVV
		BR_Vic_0044_3_2011	PX277758	DNA-B	BamHI	OxYVV
45	2011	BR_Vic_0045_1_2011	PX277614	DNA-A	EcoRV	OxYVV-S1a
46	2011	BR_Vic_0046_1_2011	PX277779	DNA-B	BamHI	OxYVV
		BR_Vic_0046_2_2011	PX277781	DNA-B	ClaI	OxYVV
47	2011	BR_Vic_0047_1_2011	PX277615	DNA-A	ClaI	OxYVV-S1c
		BR_Vic_0047_2_2011	PX277616	DNA-A	ClaI	OxYVV-S1c
104	2012	BR_Vic_0104_4_2012	PX277617	DNA-A	EcoRV	OxYVV-S1a
105	2012	BR_Vic_0105_1_2012	PX277618	DNA-A	EcoRV	OxYVV-S1a
106	2012	BR_Vic_0106_1_2012	PX277619	DNA-A	EcoRV	OxYVV-S1a
		BR_Vic_0106_2_2012	PX277620	DNA-A	EcoRV	OxYVV-S1a
107	2012	BR_Vic_0107_1_2012	PX277621	DNA-A	EcoRV	OxYVV
111	2012	BR_Vic_0111_1_2012	PX277622	DNA-A	EcoRV	OxYVV-S1a
		BR_Vic_0111_2_2012	PX277623	DNA-A	EcoRV	OxYVV-S1a
112	2012	BR_Vic_0112_1_2012	PX277624	DNA-A	EcoRV	OxYVV-S1c
114	2012	BR_Vic_0114_1_2012	PX277625	DNA-A	ClaI	OxYVV-S1b
116	2012	BR_Vic_0116_1_2012	PX277626	DNA-A	EcoRV	OxYVV-S1c

117	2012	BR_Vic_0117_1_2012	PX277627	DNA-A	EcoRV	OxYVV-S1c
119	2012	BR_Vic_0119_1_2012	PX277628	DNA-A	EcoRV	OxYVV-S1a
120	2012	BR_Vic_0120_1_2012	PX277629	DNA-A	EcoRV	OxYVV-S1a
123	2012	BR_Vic_0123_1_2012	PX277630	DNA-A	EcoRV	OxYVV-S1a
125	2012	BR_Vic_0125_1_2012	PX277800	DNA-A	EcoRV	SiYLCV
126	2012	BR_Vic_0126_1_2012	PX277631	DNA-A	EcoRV	OxYVV-S1a
		BR_Vic_0126_2_2012	PX277763	DNA-B	EcoRV	OxYVV
128	2012	BR_Vic_0128_1_2012	PX277632	DNA-A	EcoRV	OxYVV-S1a
131	2012	BR_Vic_0131_1_2012	PX277633	DNA-A	EcoRV	OxYVV-S1a
132	2012	BR_Vic_0132_1_2012	PX277634	DNA-A	EcoRV	OxYVV-S1a
133	2012	BR_Vic_0133_1_2012	PX277635	DNA-A	ClaI	OxYVV-S1a
136	2012	BR_Vic_0136_1_2012	PX277636	DNA-A	ClaI	OxYVV-S1c
201	2013	BR_Vic_0201_1_2013	PX277637	DNA-A	ClaI	OxYVV-S1c
206	2013	BR_Vic_0206_1_2013	PX277638	DNA-A	PteI	OxYVV-S1a
211	2013	BR_Vic_0211_1_2013	PX277639	DNA-A	ClaI	OxYVV-S1a
212	2013	BR_Vic_0212_1_2013	PX277640	DNA-A	BssHII	OxYVV-S1a
215	2013	BR_Vic_0215_1_2013	PX277641	DNA-A	BssHII	OxYVV-S1a
218	2013	BR_Vic_0218_1_2013	PX277642	DNA-A	ClaI	OxYVV-S1a
220	2013	BR_Vic_0220_1_2013	PX277643	DNA-A	BssHII	OxYVV-S1a
224	2013	BR_Vic_0224_4_2013	PX277871	DNA-B	BssHII	SiYLCV
225	2013	BR_Vic_0225_1_2013	PX277644	DNA-A	BssHII	OxYVV-S1a
226	2013	BR_Vic_0226_1_2013	PX277645	DNA-A	ClaI	OxYVV-S1a
227	2013	BR_Vic_0227_1_2013	PX277646	DNA-A	BssHII	OxYVV-S1a
228	2013	BR_Vic_0228_1_2013	PX277647	DNA-A	ClaI	OxYVV-S1a
229	2013	BR_Vic_0229_1_2013	PX277648	DNA-A	ClaI	OxYVV-S1a
230	2013	BR_Vic_0230_1_2013	PX277796	DNA-B	BssHII	OxYVV
230	2013	BR_Vic_0230_3_2013	PX277649	DNA-A	BssHII	OxYVV
230	2013	BR_Vic_0230_4_2013	PX277650	DNA-A	BssHII	OxYVV
231	2013	BR_Vic_0231_1_2013	PX277651	DNA-A	BssHII	OxYVV-S1c
232	2013	BR_Vic_0232_1_2013	PX277652	DNA-A	BssHII	OxYVV-S1a
233	2013	BR_Vic_0233_1_2013	PX277653	DNA-A	ClaI	OxYVV-S1a
237	2013	BR_Vic_0237_1_2013	PX277654	DAN-A	BssHII	OxYVV-S1a
253	2013	BR_Vic_0253_1_2013	PX277655	DNA-A	BssHII	OxYVV-S1a
256	2013	BR_Vic_0256_1_2013	PX277656	DNA-A	BssHII	OxYVV-S1a
301	2014	BR_Vic_0301_2_2014	PX277872	DNA-B	PteI	SiYLCV
303	2014	BR_Vic_0303_1_2014	PX277657	DNA-A	BssHII	OxYVV-S1a
		BR_Vic_0303_2_2014	PX277658	DNA-A	BssHII	OxYVV-S1a
304	2014	BR_Vic_0304_06_2014	PX277873	DNA-B	ClaI	SiYLCV

307	2014	BR_Vic_0307_1_2014	PX277659	DNA-A	BssHII	OxYVV-S1a
309	2014	BR_Vic_0309_1_2014	PX277660	DNA-A	PteI	OxYVV-S1c
310	2014	BR_Vic_0310_1_2014	PX277661	DNA-A	ClaI	OxYVV-S1a
		BR_Vic_0310_2_2014	PX277772	DNA-B	ClaI	OxYVV-S1a
311	2014	BR_Vic_0311_1_2014	PX277662	DNA-A	BssHII	OxYVV-S1a
		BR_Vic_0311_2_2014	PX277663	DNA-A	BssHII	OxYVV-S1a
312	2014	BR_Vic_0312_1_2014	PX277664	DNA-A	BssHII	OxYVV-S1a
313	2014	BR_Vic_0313_1_2014	PX277665	DNA-A	BssHII	OxYVV-S1a
315	2014	BR_Vic_0315_1_2014	PX277666	DNA-A	ClaI	OxYVV-S1a
317	2014	BR_Vic_0317_1_2014	PX277667	DNA-A	BssHII	OxYVV-S1a
318	2014	BR_Vic_0318_1_2014	PX277668	DNA-A	BssHII	OxYVV-S1a
319	2014	BR_Vic_0319_1_2014	PX277669	DNA-A	BssHII	OxYVV-S1a
320	2014	BR_Vic_0320_1_2014	PX277670	DNA-A	BssHII	OxYVV-S1a
321	2014	BR_Vic_0321_1_2014	PX277671	DNA-A	BssHII	OxYVV-S1a
323	2014	BR_Vic_0323_6_2014	PX277789	DNA-B	ClaI	OxYVV-S1a
		BR_Vic_0323_1_2014	PX277672	DNA-A	ClaI	OxYVV-S1a
		BR_Vic_323_06_2014	PX277790	DNA-B	ClaI	OxYVV
325	2014	BR_Vic_0325_1_2014	PX277673	DNA-A	PteI	OxYVV-S1a
326	2014	BR_Vic_0326_1_2014	PX277674	DNA-A	BssHII	OxYVV-S1a
		BR_Vic_0326_2_2014	PX277675	DNA-A	BssHII	OxYVV-S1a
327	2014	BR_Vic_0327_1_2014	PX277676	DNA-A	BssHII	OxYVV-S1a
329	2014	BR_Vic_0329_1_2014	PX277677	DNA-A	BssHII	OxYVV-S1a
331	2014	BR_Vic_0331_1_2014	PX277678	DNA-A	PteI	OxYVV-S1a
334	2014	BR_Vic_0334_1_2014	PX277679	DNA-A	EcoRV	OxYVV-S1a
335	2014	BR_Vic_0335_1_2014	PX277680	DNA-A	PteI	OxYVV-S1a
337	2014	BR_Vic_0337_1_2014	PX277681	DNA-A	PteI	OxYVV-S1a
338	2014	BR_Vic_0338_1_2014	PX277682	DNA-A	PteI	OxYVV-S1a
401	2016	BR_Vic_0401_1_2016	PX277683	DNA-A	BssHII	OxYVV-S1b
403	2016	BR_Vic_0403_1_2016	PX277809	DNA-A	BssHII	SIYLCV
		BR_Vic_0403_2_2016	PX277810	DNA-A	BssHII	SIYLCV
404	2016	BR_Vic_0404_3_2016	PX277875	DNA-B	BssHII	SiYLCV
		BR_Vic_0404_09_2016	PX277874	DNA-B	BssHII	SiYLCV
405	2016	BR_Vic_0405_1_2016	PX277684	DNA-A	PteI	OxYVV-S1b
		BR_Vic_0405_2_2016	PX277685	DNA-A	PteI	OxYVV-S1b
406	2016	BR_Vic_0406_1_2016	PX277686	DNA-A	BssHII	OxYVV-S1b
407	2016	BR_Vic_0407_1_2016	PX277687	DNA-A	ClaI	OxYVV-S1b
408	2016	BR_Vic_0408_1_2016	PX277688	DNA-A	PteI	OxYVV-S1b
409	2016	BR_Vic_0409_1_2016	PX277689	DNA-A	PteI	OxYVV-S1a

		BR_Vic_0409_2_2016	PX277690	DNA-A	PteI	OxYVV-S1a
		BR_Vic_0409_3_2016	PX277691	DNA-A	PteI	OxYVV-S1a
410	2019	BR_Vic_0410_1_2016	PX277692	DNA-A	PteI	OxYVV-S1a
411	2016	BR_Vic_0411_3_2016	PX277876	DNA-B	PteI	SiYLCV
412	2016	BR_Vic_0412_1_2016	PX277828	DNA-A	EcoRV	SiYLCV
414	2016	BR_Vic_0414_1_2016	PX277841	DNA-A	PteI	SiYLCV
	2016	BR_Vic_0414_16_2016	PX277877	DNA-B	PteI	n.d.
415	2016	BR_Vic_0415_1_2016	PX277811	DNA-A	BssHII	SiYLCV
		BR_Vic_415_07_2016	PX277913	DNA-B	BssHII	SiYLCV
416	2016	BR_Vic_0416_1_2016	PX277829	DNA-A	BssHII	SiYLCV
		BR_Vic_0416_2_2016	PX277830	DNA-A	PteI	SiYLCV
418	2016	BR_Vic_0418_2_2016	PX277878	DNA-B	BssHII	SiYLCV
419	2016	BR_Vic_0419_07_2016	PX277879	DNA-B	PteI	SiYLCV
		BR_Vic_0419_5_2016	PX277880	DNA-B	PteI	SiYLCV
421	2016	BR_Vic_0421_1_2016	PX277842	DNA-A	EcoRV	SiYLCV
423	2016	BR_Vic_0423_1_2016	PX277807	DNA-A	PteI	SiYLCV
		BR_Vic_0423_4_2016	PX277881	DNA-B	PteI	SiYLCV
426	2016	BR_Vic_0426_1_2016	PX277837	DNA-A	EcoRV	SiYLCV
428	2016	BR_Vic_0428_1_2016	PX277840	DNA-A	PteI	SiYLCV
		BR_Vic_0428_2_2016	PX277832	DNA-A	PteI	SiYLCV
429	2016	BR_Vic_0429_1_2016	PX277813	DNA-A	BssHII	SiYLCV
		BR_Vic_0429_2_2016	PX277831	DNA-A	BssHII	SiYLCV
501	2017	BR_Vic_0501_1_2016	PX277844	DNA-A	PteI	SiYLCV
502	2017	BR_Vic_0502_1_2017	PX277812	DNA-A	PteI	SiYLCV
504	2017	BR_Vic_0504_1_2017	PX277822	DNA-A	PteI	SiYLCV
505	2017	BR_Vic_0505_1_2017	PX277847	DNA-A	PteI	SiYLCV
		BR_Vic_0505_2_2017	PX277882	DNA-B	PteI	SiYLCV
		BR_Vic_0505_7_2017	PX316469	DNA-A	PteI	SiYLCV
506	2017	BR_Vic_0506_1_2017	PX277843	DNA-A	BssHII	SiYLCV
		BR_Vic_0506_1_2017	PX277883	DNA-B	BssHII	SiYLCV
507	2017	BR_Vic_0507_1_2017	PX277823	DNA-A	PteI	SiYLCV
508	2017	BR_Vic_0508_1_2017	PX277693	DNA-A	EcoRV	OxYVV-S1a
510	2017	BR_Vic_0510_2_2017	PX277884	DNA-B	PteI	SiYLCV
511	2017	BR_Vic_0511_1_2017	PX277814	DNA-A	PteI	SiYLCV
512	2017	BR_Vic_0512_1_2017	PX277808	DNA-A	PteI	SiYLCV
		BR_Vic_0512_5_2017	PX316470	DNA-A	PteI	SiYLCV
513	2017	BR_Vic_0513_4_2017	PX277694	DNA-A	EcoRV	OxYVV-S1a
514	2017	BR_Vic_0514_1_2017	PX277695	DNA-A	PteI	OxYVV-S1b

515	2017	BR_Vic_0515_1_2017	PX277696	DNA-A	PteI	OxYVV-S1a
516	2017	BR_Vic_0516_1_2017	PX277845	DNA-A	EcoRV	SiYLCV
		BR_Vic_0516_2_2017	PX277846	DNA-A	EcoRV	SiYLCV
517	2017	BR_Vic_0517_1_2017	PX277697	DNA-A	PteI	OxYVV-S1a
**518**	2017	BR_Vic_0518_1_2017	PX277885	DNA-A	PteI	OxYVV-S1a
		BR_Vic_0518_1_2017	PX277698	DNA-A	PteI	SiYLCV
520	2017	BR_Vic_0520_1_2017	PX277699	DNA-A	PteI	OxYVV-S1a
522	2017	BR_Vic_0522_1_2017	PX277700	DNA-A	PteI	OxYVV-S1a
		BR_Vic_0522_2_2017	PX277701	DNA-A	PteI	OxYVV-S1a
524	2017	BR_Vic_0524_1_2017	PX277702	DNA-A	PteI	OxYVV-S1a
525	2017	BR_Vic_0525_1_2017	PX289945	DNA-B	PteI	n.d.
**527**	2017	BR_Vic_0527_1_2017	PX277824	DNA-A	PteI	SiYLCV
		BR_Vic_0527_2_2017	PX277703	DNA-A	PteI	OxYVV-S1a
529	2017	BR_Vic_0529_1_2017	PX277704	DNA-A	PteI	OxYVV-S1a
530	2017	BR_Vic_0530_1_2017	PX277801	DNA-A	PteI	SiYLCV
531	2017	BR_Vic_0531_1_2017	PX277803	DNA-A	EcoRV	SiYLCV
603	2018	BR_Vic_0603_1_2018	PX277821	DNA-A	PteI	SiYLCV
		BR_Vic_0603_5_2018	PX277886	DNA-B	PteI	SiYLCV
604	2018	BR_Vic_0604_1_2018	PX277827	DNA-A	PteI	SiYLCV
607	2018	BR_Vic_0607_07_2018	PX246749	DNA-B	PteI	SimMV
608	2018	BR_Vic_0608_1_2018	PX277569	DNA-A	PteI	SimMV-a
610	2018	BR_Vic_0610_1_2018	PX277839	DNA-A	PteI	SiYLCV
		BR_Vic_0610_1_2018	PX277887	DNA-B	PteI	SiYLCV
612	2018	BR_Vic_0612_07_2018	PX277888	DNA-B	PteI	SiYLCV
615	2018	BR_Vic_0615_1_2018	PX277836	DNA-A	PteI	SiYLCV
618	2018	BR_Vic_0618_1_2018	PX277573	DNA-A	PteI	SimMV-a
		BR_Vic_0618_2_2018	PX277574	DNA-A	PteI	SimMV-a
619	2018	BR_Vic_0619_1_2018	PX277705	DNA-A	PteI	OxYVV-S1a
620	2018	BR_Vic_0620_1_2018	PX277706	DNA-A	PteI	OxYVV-S1a
622	2018	BR_Vic_0622_2_2018	PX277889	DNA-B	PteI	SiYLCV
		BR_Vic_0622_1_2018	PX277802	DNA-A	PteI	SiYLCV
		BR_Vic_0622_24_2018	PX277890	DNA-B	PteI	SiYLCV
623	2018	BR_Vic_0623_1_2018	PX277707	DNA-A	PteI	OxYVV-S1a
624	2018	BR_Vic_0624_1_2018	PX277708	DNA-A	PteI	OxYVV-S1a
625	2018	BR_Vic_0625_1_2018	PX277709	DNA-A	PteI	OxYVV-S1a
626	2018	BR_Vic_0626_1_2018	PX277849	DNA-A	PteI	SiYLCV
		BR_Vic_0626_2_2018	PX277850	DNA-A	PteI	SiYLCV
		BR_Vic_0626_3_2018	PX277851	DNA-A	PteI	SiYLCV

627	2018	BR_Vic_0627_2018	PX277580	DNA-A	PteI	SimMV-b
628	2018	BR_Vic_0628_1_2018	PX277582	DNA-A	PteI	SimMV-b
630	2018	BR_Vic_0630_1_2018	PX277710	DNA-A	PteI	OxYVV-S1a
631	2018	BR_Vic_0631_1_2018	PX277711	DNA-A	PteI	OxYVV-S1a
632	2018	BR_Vic_0632_27_2018	PX289942	DNA-B	PteI	SiMV
634	2018	BR_Vic_0634_1_2018	PX277712	DNA-A	PteI	OxYVV-S1a
636	2018	BR_Vic_0636_1_2018	PX277713	DNA-A	PteI	OxYVV-S1a
637	2018	BR_Vic_0637_1_2018	PX277805	DNA-A	PteI	SiYLCV
		BR_Vic_0637_2_2018	PX277891	DNA-B	PteI	SiYLCV
639	2018	BR_Vic_0639_1_2018	PX277577	DNA-A	PteI	SimMV-a
701	2019	BR_Vic_0701_4_2019	PX277892	DNA-B	BssHII	SiYLCV
702	2019	BR_Vic_0702_5_2019	PX277825	DNA-A	XhoI	SiYLCV
		BR_Vic_0702_8_2019	PX277826	DNA-A	XhoI	SiYLCV
703	2019	BR_Vic_0703_10_2019	PX277893	DNA-B	BssHII	SiYLCV
704	2019	BR_Vic_0704_5_2019	PX277833	DNA-A	EcoRV	SiYLVC
		BR_Vic_0704_13_2019	PX277894	DNA-B	BssHII	SiYLVC
705	2019	BR_Vic_0705_1_2019	PX277895	DNA-B	BamHI	SiYLCV
		BR_Vic_0705_2_2019	PX277896	DNA-B	BamHI	SiYLCV
706	2019	BR_Vic_0706_4_2019	PX277834	DNA-A	PteI	SiYLCV
707	2019	BR_Vic_0707_07_2019	PX277897	DNA-B	BssHII	SiYLCV
708	2019	BR_Vic_0708_2_2019	PX277848	DNA-B	BssHII	SiYLCV
**710**	2019	BR_Vic_0710_3_2019	PX277714	DNA-A	BssHII	OxYVV-S1a
		BR_Vic_0710_9_2019	PX277715	DNA-A	BssHII	OxYVV-S1a
		BR_Vic_0710_10_2019	PX277575	DNA-A	BssHII	SimMV-a
712	2019	BR_Vic_0712_1_2019	PX277576	DNA-A	BssHII	SimMV-a
		BR_Vic_0712_4_2019	PX246747	DNA-B	ClaI	SimMV
713	2019	BR_Vic_0713_9_2019	PX277852	DNA-A	BssHII	SiYLCV
714	2019	BR_Vic_0714_1_2019	PX277854	DNA-A	BssHII	SiYLCV
		BR_Vic_0714_8_2019	PX316471	DNA-A	BssHII	SiYLCV
715	2019	BR_Vic_0715_12_2019	PX277856	DNA-A	BssHII	SiYLCV
716	2019	BR_Vic_0716_15_2019	PX277853	DNA-A	BssHII	SiYLCV
718	2019	BR_Vic_0718_2_2019	PX277716	DNA-A	BssHII	OxYVV-S1a
		BR_Vic_0718_8_2019	PX277717	DNA-A	BssHII	OxYVV-S1a
719	2019	BR_Vic_0719_8_2019	PX277718	DNA-A	BssHII	OxYVV-S1a
		BR_Vic_0719_2_2019	PX277794	DNA-B	BssHII	OxYVV
720	2019	BR_Vic_0720_1_2019	PX277581	DNA-A	BssHII	SimMV-b
723	2019	BR_Vic_0723_8_2019	PX277855	DNA-A	BssHII	SIYLCV
724	2019	BR_Vic_0724_6_2019	PX277719	DNA-A	BssHII	OxYVV-S1a

**725**	2019	BR_Vic_0725_6_2019	PX277720	DNA-A	BssHII	OxYVV-S1a
		BR_Vic_0725_3_2019	PX277898	DNA-B	BssHII	SiYLCV
726	2019	BR_Vic_0726_06_2019	PX246748	DNA-B	BssHII	SimM
736	2019	BR_Vic_0736_2_2019	PX277578	DNA-A	EcoRV	SimMV-a
741	2019	BR_Vic_0741_4_2019	PX277721	DNA-A	BssHII	OxYVV-S1a
742	2019	BR_Vic_0742_4_2019	PX277857	DNA-A	BssHII	SiYLCV
801	2020	BR_Vic_0801_1_2020	PX277722	DNA-A	ClaI	OxYVV-S1a
		BR_Vic_0801_3_2020	PX277791	DNA-B	ClaI	OxYVV
802	2020	BR_Vic_0802_9_2020	PX277815	DNA-A	BssHII	SiYLCV
**803**	2020	BR_Vic_0803_3_2020	PX277570	DNA-A	EcoRV	SimMV-a
		BR_Vic_0803_4_2020	PX277571	DNA-A	EcoRV	SimMV-a
		BR_Vic_0803_1_2020	PX277899	DNA-B	BssHII	SiYLCV
		BR_Vic_0803_5_2020	PX246744	DNA-B	BssHII	SimMV
		BR_Vic_0803_2_2020	PX246745	DNA-B	EcoRV	SimMV
		BR_Vic_0803_08_2020	PX246746	DNA-B	EcoRV	SimMV
804	2020	BR_Vic_0804_1_2020	PX277817	DNA-A	BssHII	SiYLCV
		BR_Vic_0804_5_2020	PX277819	DNA-A	BssHII	SiYLCV
		BR_Vic_0804_8_2020	PX277820	DNA-A	BssHII	SiYLCV
		BR_Vic_0804_2_2020	PX277900	DNA-B	ClaI	SiYLCV
805	2020	BR_Vic_0805_3_2020	PX277792	DNA-B	ClaI	OxYVV
807	2020	BR_Vic_0807_1_2020	PX277858	DNA-A	BssHII	SiYLCV
808	2020	BR_Vic_0808_1_2020	PX277723	DNA-A	BssHII	OxYVV-S1b
809	2020	BR_Vic_0809_1_2020	PX277724	DNA-A	BssHII	OxYVV-S1e
810	2020	BR_Vic_0810_1_2020	PX277583	DNA-A	BssHII	SimMV-b
		BR_Vic_0810_2_2020	PX277584	DNA-A	BssHII	SimMV-b
812	2020	BR_Vic_0812_10_2020	PX277816	DNA-A	BssHII	SiYLCV
		BR_Vic_0812_2_2020	PX277901	DNA-B	ClaI	SiYLCV
814	2020	BR_Vic_0814_1_2020	PX277585	DNA-A	BssHII	SimMV-b
815	2020	BR_Vic_0815_06_2020	PX289943	DNA-B	ClaI	SiMV
816	2020	BR_Vic_0816_3_2020	PX277726	DNA-A	ClaI	OxYVV-S1a
		BR_Vic_0816_1_2020	PX277725	DNA-A	EcoRV	OxYVV-S1a
		BR_Vic_0816_3_2020	PX277765	DNA-B	EcoRV	OxYVV
818	2020	BR_Vic_0818_1_2020	PX277838	DNA-A	EcoRV	SiYLCV
		BR_Vic_0818_8_2020	PX277818	DNA-A	EcoRV	SiYLCV
		BR_Vic_0818_3_2020	PX277835	DNA-A	BssHII	SiYLCV
819	2020	BR_Vic_0819_9_2020	PX277859	DNA-A	BssHII	SiYLCV
831	2020	BR_Vic_0831_1_2020	PX277902	DNA-B	ClaI	SiYLCV
		BR_Vic_0831_2_2020	PX316472	DNA-A	ClaI	SiYLCV
832	2020	BR_Vic_0832_1_2020	PX277727	DNA-A	EcoRV	OxYVV-S1b

		BR_Vic_0832_2_2020	PX277728	DNA-A	EcoRV	OxYVV-S1b
		BR_Vic_0832_4_2020	PX277729	DNA-A	ClaI	OxYVV-S1b
		BR_Vic_0832_1_2020	PX277783	DNA-B	ClaI	OxYVV
		BR_Vic_0832_2_2020	PX277784	DNA-B	ClaI	OxYVV
836	2020	BR_Vic_0836_07_2020	PX246743	DNA-B	BssHII	SimMV
837	2020	BR_Vic_0837_5_2020	PX277572	DNA-A	EcoRV	SimMV-a
		BR_Vic_0837_1_2020	PX246742	DNA-B	BssHII	SimMV
		BR_Vic_0837_13_2020	PX246741	DNA-B	BssHII	SimMV
839	2020	BR_Vic_0839_1_2020	PX277860	DNA-A	EcoRV	SiYLCV
		BR_Vic_0839_3_2020	PX277861	DNA-A	EcoRV	SiYLCV
901	2021	BR_Vic_0901_1_2021	PX277730	DNA-A	BssHII	OxYVV-S2
		BR_Vic_0901_1_2021	PX277797	DNA-B	BamHI	OxYVV
		BR_Vic_0901_2_2021	PX277798	DNA-B	BamHI	OxYVV
**902**	2021	BR_Vic_0902_6_2021	PX277731	DNA-A	BssHII	OxYVV-S2
		BR_Vic_0902_10_2021	PX277903	DNA-B	BssHII	SiYLCV
903	2021	BR_Vic_0903_1_2021	PX277732	DNA-A	BssHII	OxYVV-S1a
904	2021	BR_Vic_0904_10_2021	PX277733	DNA-A	ClaI	OxYVV-S1a
**905**	2021	BR_Vic_0905_1_2021	PX277734	DNA-A	EcoRV	OxYVV-S1a
		BR_Vic_0905_2_2021	PX277904	DNA-B	ClaI	SiYLCV
		BR_Vic_0905_8_2021	PX316473	DNA-A	ClaI	SiYLCV
907	2021	BR_Vic_0907_6_2021	PX277868	DNA-A	BssHII	SiYLCV
		BR_Vic_0907_8_2021	PX277869	DNA-A	BssHII	SiYLCV
908	2021	BR_Vic_0908_2_2021	PX277735	DNA-A	BssHII	OxYVV-S2
909	2021	BR_Vic_0909_8_2021	PX277736	DNA-A	EcoRV	OxYVV-S2
910	2021	BR_Vic_0910_7_2021	PX277737	DNA-A	BssHII	OxYVV-S2
911	2021	BR_Vic_0911_4_2021	PX289944	DNA-A	EcoRV	MaYVV
912	2021	BR_Vic_0912_10_2021	PX289941	DNA-A	ClaI	SiMV
		BR_Vic_0912_9_2021	PX289936	DNA-B	ClaI	SiMV
913	2021	BR_Vic_0913_4_2021	PX277579	DNA-A	ClaI	SimMV
917	2021	BR_Vic_0917_2_2021	PX277738	DNA-A	BssHII	OxYVV-S1a
		BR_Vic_0917_1_2021	PX277799	DNA-B	ClaI	OxYVV
918	2021	BR_Vic_0918_5_2021	PX277804	DNA-A	BssHII	SiYLCV
919	2021	BR_Vic_0919_1_2021	PX277867	DNA-A	BssHII	SiYLCV
921	2021	BR_Vic_0921_1_2021	PX289937	DNA-A	BssHII	SiMV
923	2021	BR_Vic_0923_8_2021	PX277862	DNA-A	BssHII	SiYLCV
		BR_Vic_0923_07_2021	PX277905	DNA-B	BssHII	SiYLCV
924	2021	BR_Vic_0924_1_2021	PX289938	DNA-A	EcoRV	SiMV
		BR_Vic_0924_6_2021	PX289939	DNA-A	EcoRV	SiMV
925	2021	BR_Vic_0925_1_2021	PX277906	DNA-B	BssHII	SiYLCV

926	2021	BR_Vic_0926_6_2021	PX277806	DNA-A	BssHII	SiYLCV
		BR_Vic_0926_2_2021	PX277908	DNA_B	BssHII	SiYLCV
		BR_Vic_0926_5_2021	PX277909	DNA-B	BssHII	SiYLCV
		BR_Vic_0926_10_2021	PX277907	DNA-B	BssHII	SiYLCV
928	2021	BR_Vic_0928_2_2021	PX277910	DNA-B	BssHII	SiYLCV
1001	2022	BR_Vic_1001_1_2022	PX277864	DNA-A	EcoRV	SiYLCV
1002	2022	BR_Vic_1002_2_2022	PX277911	DNA-B	EcoRV	SiYLCV
1004	2022	BR_Vic_1004_3_2022	PX277863	DNA-A	EcoRV	SiYLCV
1006	2022	BR_Vic_1006_1_2022	PX277739	DNA-A	EcoRV	OxYVV-S1a
1008	2022	BR_Vic_1008_3_2022	PX277870	DNA-A	EcoRV	SiYLCV
**1009**	2022	BR_Vic_1009_3_2022	PX277740	DNA-A	EcoRV	OxYVV-S1a
		BR_Vic_1009_5_2022	PX277741	DNA-A	EcoRV	OxYVV-S1a
		BR_Vic_1009_1_2022	PX277912	DNA-B	EcoRV	SiYLCV
		BR_Vic_1009_2_2022	PX316474	DNA-A	EcoRV	SiYLCV
1010	2022	BR_Vic_1010_1_2022	PX277865	DNA-A	EcoRV	SiYLCV
1012	2022	BR_Vic_1012_1_2022	PX277742	DNA-A	EcoRV	OxYVV-S1d
		BR_Vic_1012_2_2022	PX277764	DNA-B	EcoRV	OxYVV
1013	2022	BR_Vic_1013_1_2022	PX277743	DNA-A	EcoRV	OxYVV-S1d
1014	2022	BR_Vic_1014_5_2022	PX277744	DNA-A	EcoRV	OxYVV-S1d
		BR_Vic_1014_06_2022	PX277767	DNA-B	EcoRV	OxYVV
1015	2022	BR_Vic_1015_2022	PX277866	DNA-A	EcoRV	SiYLCV
1016	2022	BR_Vic_1016_2_2022	PX277745	DNA-A	EcoRV	OxYVV-S1d
1018	2022	BR_Vic_1018_8_2022	PX277746	DNA-A	EcoRV	OxYVV-S1a
1020	2022	BR_Vic_1020_2_2022	PX277747	DNA-A	EcoRV	OxYVV-S1a
		BR_Vic_1020_3_2022	PX277766	DNA-B	EcoRV	OxYVV
**1021**	2022	BR_Vic_1021_8_2022	PX289940	DNA-A	EcoRV	SiMV
		BR_Vic_1021_10_2022	PX277768	DNA-B	EcoRV	OxYVV

Supplementary Table S2. Number of DNA-A clones of each begomovirus and their respective variants obtained from *Sida acuta* samples collected in Viçosa, MG, from 2011 to 2022.

**Species-variant**	**2011**	**2012**	**2013**	**2014**	**2016**	**2017**	**2018**	**2019**	**2020**	**2021**	**2022**	**Total**
OxYVV-S1a	19	15	17	25	4	11	9	8	3	3	4	118
OxYVV-S1b	2	1	0	0	6	1	0	0	4	0	0	14
OxYVV-S1c	10	4	3	1	0	0	0	0	0	0	0	18
OxYVV-S1d	0	0	0	0	0	0	0	0	0	0	4	4
OxYVV-S1e	0	0	0	0	0	0	0	0	1	0	0	1
OxYVV-S2	0	0	0	0	0	0	0	0	0	6	1	7
SiYLCV	0	1	0	0	14	15	9	12	13	7	6	77
SiMMV-a	0	0	0	0	0	0	4	3	3	1	0	11
SiMMV-b	0	0	0	0	0	0	2	1	3	0	0	6
SiMV	0	0	0	0	0	0	0	0	0	4	1	5
MaYSV	0	0	0	0	0	0	0	0	0	1	0	1
Total	31	21	20	26	24	27	24	24	27	22	16	262

Supplementary Table S3. Distribution of single and mixed begomovirus infections detected in *Sida acuta* plants sampled in the same area from 2011 to 2022. Infection categories are based on DNA-A sequence identification and restriction fragment patterns obtained after MspI digestion of RCA-amplified genomes. "nd" indicates viral components for which the cognate DNA-A was not available, and therefore the species and variant could not be determined. Mixed infections were confirmed by the recovery of distinct clones displaying different MspI restriction profiles from the same plant sample.

Virus/Strain/Variant	2011	2012	2013	2014	2016	2017	2018	2019	2020	2021	2022
Single Infection											
OxYVV nd	7	1	0	0	0	0	0	0	1	0	0
OxYVV-S1a	13	12	16	22	2	8	9	4	2	3	3
OxYVV-S1b	1	1	0	0	5	1	0	0	2	0	0
OxYVV-S1c	5	3	2	1	0	0	0	0	0	0	0
OxYVV-S1d	0	0	0	0	0	0	0	0	0	0	3
OxYVV-S1e	0	0	0	0	0	0	0	0	1	0	0
OxYVV-S2	0	0	0	0	0	0	0	0	0	4	0
SiYLCV	0	1	0	0	13	12	8	13	7	7	6
SIYLCV nd	0	0	1	2	1	0	0	1	0	0	0
nd	0	0	0	0	0	1	0	0	0	0	0
SiMV	0	0	0	0	0	0	1	0	1	3	1
SimMV-a	0	0	0	0	0	0	3	2	2	0	0
SimMV-b	0	0	0	0	0	0	2	1	2	0	0
SimMV nd	0	0	0	0	0	0	1	1	1	1	0
MaYVV	0	0	0	0	0	0	0	0	0	1	0
Mixed Infection											
OxYVV-S1a and OxYVV-S1b	2	1	1	0	0	0	0	0	0	0	0
OxYVV-S1A and SiYLCV	0	0	0	0	0	1	1	1	0	2	1
OxYVV-S1a and SimMV-a	0	0	0	0	0	0	0	1	0	0	0

SimMV-A and SiYLCV	0	0	0	0	0	0	0	0	1	0	0
OxYVV-S1A and SiMV	0	0	0	0	0	0	0	0	0	0	1

Supplementary Table S4. Proportion of non-synonymous to synonymous substitutions for DNA-A genes of the begomoviruses infecting *Sida acuta* in Viçosa, MG, and sites under positive or negative selection according to three maximum likelihood-based methods.

Gene	Tajima's D	Fu & Li's		dN/dS	SLAC		MEME		FUBAR	
		F*	***D****		**Positive**	**Negative**	**Positive**	**Negative**	**Positive**	**Negative**
**OxYVV**										
CP	-1.351	-1.609	-1.387	0.129	-	18, 25, 34, 37, 71, 72 86, 94, 137, 149 154, 156, 177, 209, 217, 225 245, 249	31	-	-	18, 20, 25, 32, 34, 37, 47, 49, 50, 53, 71, 72, 73, 83, 86, 94, 113, 137, 149, 154, 156, 158, 173, 177, 182, 191, 209, 217, 218, 223, 224, 225, 237, 245, 249
Rep	-1.356	-2.458*	-2.673*	0.259	-	40, 70, 72, 78, 116, 144, 151, 157, 186, 187, 220, 251, 274, 321, 324	60, 98, 123, 214	-	-	17, 39, 40, 62, 70, 72, 78, 110, 115, 116, 126, 143, 144, 151, 157, 166, 178, 184, 186, 187, 194, 220, 223, 235, 251, 260, 274, 314, 321, 324, 332, 347
TrAP	-1.396	-2.904*	-3.201*	0.534	-	2, 15, 121	92, 94	-	92, 119	2, 15, 33, 64, 121
Ren	-1.264	-2.216	-2.324	0.379	-	35, 47, 73, 74, 103	126	-	-	27, 31, 35, 42, 47, 73, 74 102, 103, 122,
AC4	-1.723	-1.048	-1.611	1.350	-	58	6, 19	-	9, 17, 75	58, 81
MP	1.103	0.843	0.465	0.113	-	20, 159, 182	233	-	-	159, 182, 20, 179, 71, 215, 123, 66, 120, 124, 220, 62, 129, 260, 10, 151, 35, 14, 63, 84, 85, 112, 161, 203, 97, 171, 206, 278, 291, 277, 140, 2, 180, 82, 148, 139, 193
NSP	0.562	0.051	-0.272	0.275	-	48, 115	120, 160	-	120	48, 63, 95, 110, 115, 129, 133, 145, 215

				**SiYLCV**					
CP	0.362	-0.563	-1.007	0.151	-	80, 94, 155,136	81	-	10	49, 80, 83, 90, 94, 127, 136, 155, 182, 190, 222, 239
Rep	-1.128	-1.654	-1.543	0.468	-	52, 125	172	-	44, 172, 181	12, 23, 43, 52, 53, 57, 125, 187, 259, 332
TrAP	-1.927*	-2.418	-2.082	0.573	-	-	-	-	14, 129	34, 42, 99, 103
Ren	-1.613*	-1.353	-0.831	0.319	-	-	-	-	-	122, 130
AC4	-1.876*	-1.787	-1.289	1.130	-	-	-	-	-	-
MP	0.523	0.897	0.908	0.154	-	41, 43, 66, 149, 162	32	-	-	7, 14, 20, 41, 43, 58, 66, 75, 82, 94, 113, 126, 127, 137, 149, 162, 170, 172, 184, 202, 211, 217
NSP	-1.007	-0.765	-0.441	0.319	-	201	-	-	-	160, 174, 186, 199, 201, 211

Supplementary Table S5. Distribution of nucleotide substitutions mapped to each coding region (Rep, Trap, Ren, AC4, CP, MP and NSP) of the three dominant viruses detected in this study (Oxalis yellow mosaic virus, OxYVV; Sida micrantha mosaic virus, SimMV; and Sida yellow leaf curl virus, SiYLCV). The table presents the number of substitutions by codon position relative to the species-specific consensus sequence. Substitutions were classified as synonymous or nonsynonymous. The table also shows the percentage of substitutions at each codon position, as well as the percentages of synonymous and nonsynonymous substitutions.

Gene	total_nt_diffs	pos1	pos2	pos3	%_pos1	%_pos2	%_pos3	codon_changes	syn	nonsyn	%_syn	%_nonsyn
OxYVV_AC4	407	16	246	145	3.93	60.44	35.62	386	95	291	24.61	75.38
OxYVV_Cp	1221	69	79	1073	5.65	6.47	87.87	1167	1047	120	89.71	10.28
OxYVV_Ren	911	148	282	481	16.24	30.95	52.79	839	419	420	49.94	50.05
OxYVV_Rep	2213	653	172	1388	29.50	7.77	62.72	1978	1168	810	59.04	40.95
OxYVV_Trap	557	268	58	231	48.11	10.41	41.47	448	166	282	37.05	62.94
OxYVV_MP	1242	168	142	932	13.52	11.43	75.04	1197	934	263	78.02	21.97
OxYVV_NSP	1301	254	234	813	19.52	17.98	62.49	1209	769	440	63.60	36.39
SiMMV_AC4	217	23	123	71	10.59	56.68	32.71	196	56	140	28.57	71.42
SiMMV_CP	214	27	4	183	12.61	1.86	85.51	209	188	21	89.95	10.04
SiMMV_Rep	728	209	83	436	28.70	11.40	59.89	609	378	231	62.06	37.93
SiMMV_Ren	80	15	18	47	18.75	22.5	58.75	80	56	24	70	30
SiMMV_Trap	76	21	12	43	27.63	15.78	56.57	76	46	30	60.52	39.47
SiMMV_MP	175	31	13	131	17.71	7.42	74.85	164	127	37	77.43	22.56
SiMMV_NSP	240	42	32	166	17.5	13.33	69.16	233	173	60	74.24	25.75
SiYLCV_AC4	59	9	36	14	15.25	61.01	23.72	58	13	45	22.41	77.58
SiYLCV_Cp	750	85	42	623	11.33	5.6	83.06	692	592	100	85.54	14.45
SiYLCV_Ren	195	72	26	97	36.92	13.33	49.74	184	67	117	36.41	63.58
SiYLCV_Rep	358	96	47	215	26.81	13.12	60.05	349	211	138	60.45	39.54
SiYLCV_Trap	104	33	16	55	31.73	15.38	52.88	104	55	49	52.88	47.11
SiYLCV_MP	825	95	76	654	11.51	9.21	79.27	736	565	171	76.76	23.23
SiYLCV_NSP	226	39	35	152	17.25	15.48	67.25	226	136	90	60.17	39.82

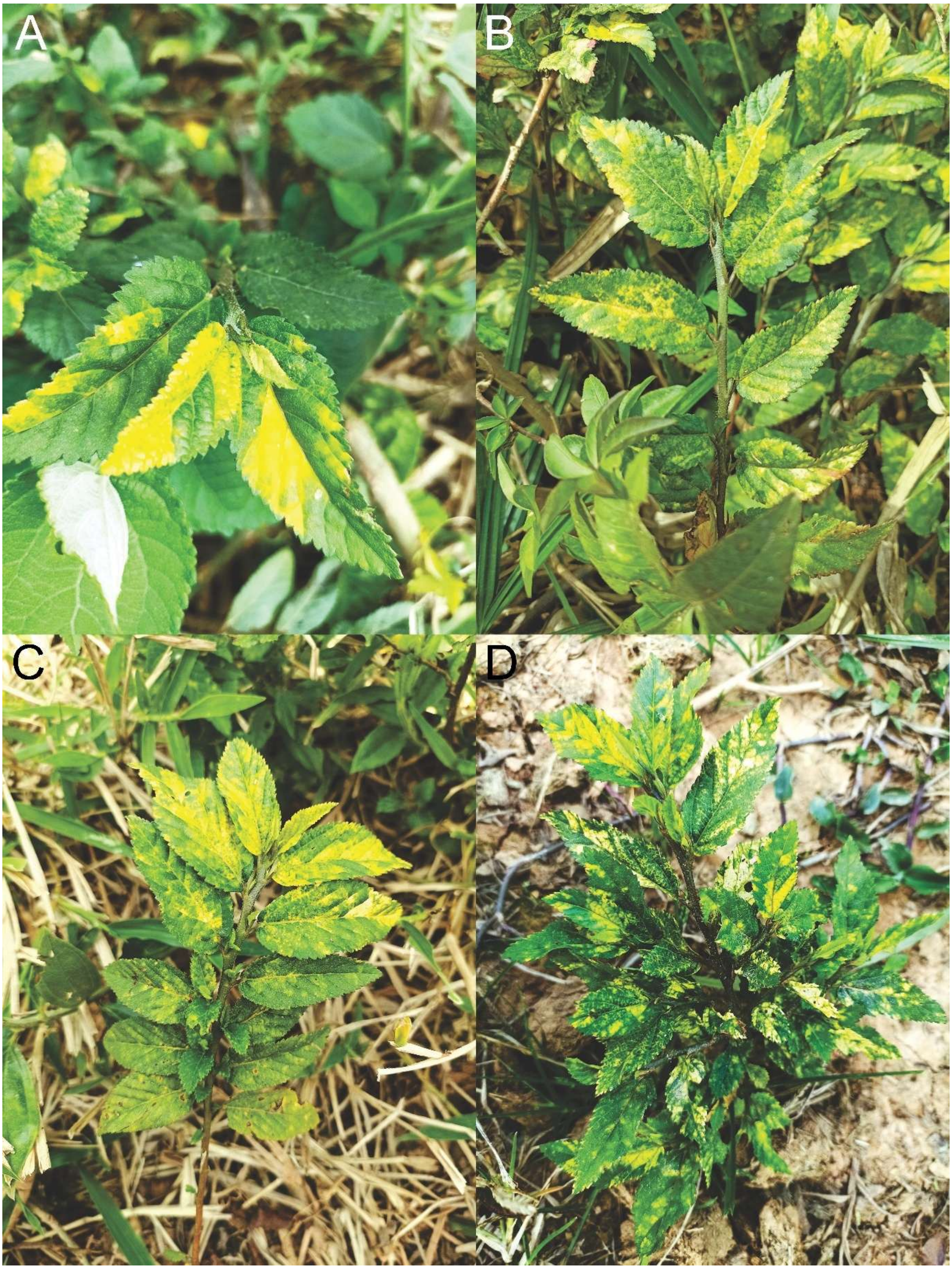

Suppl. Figure S1. *Sida acuta* plants growing in a suburban area near Viçosa, MG, Brazil, displaying symptoms of begomovirus infection.

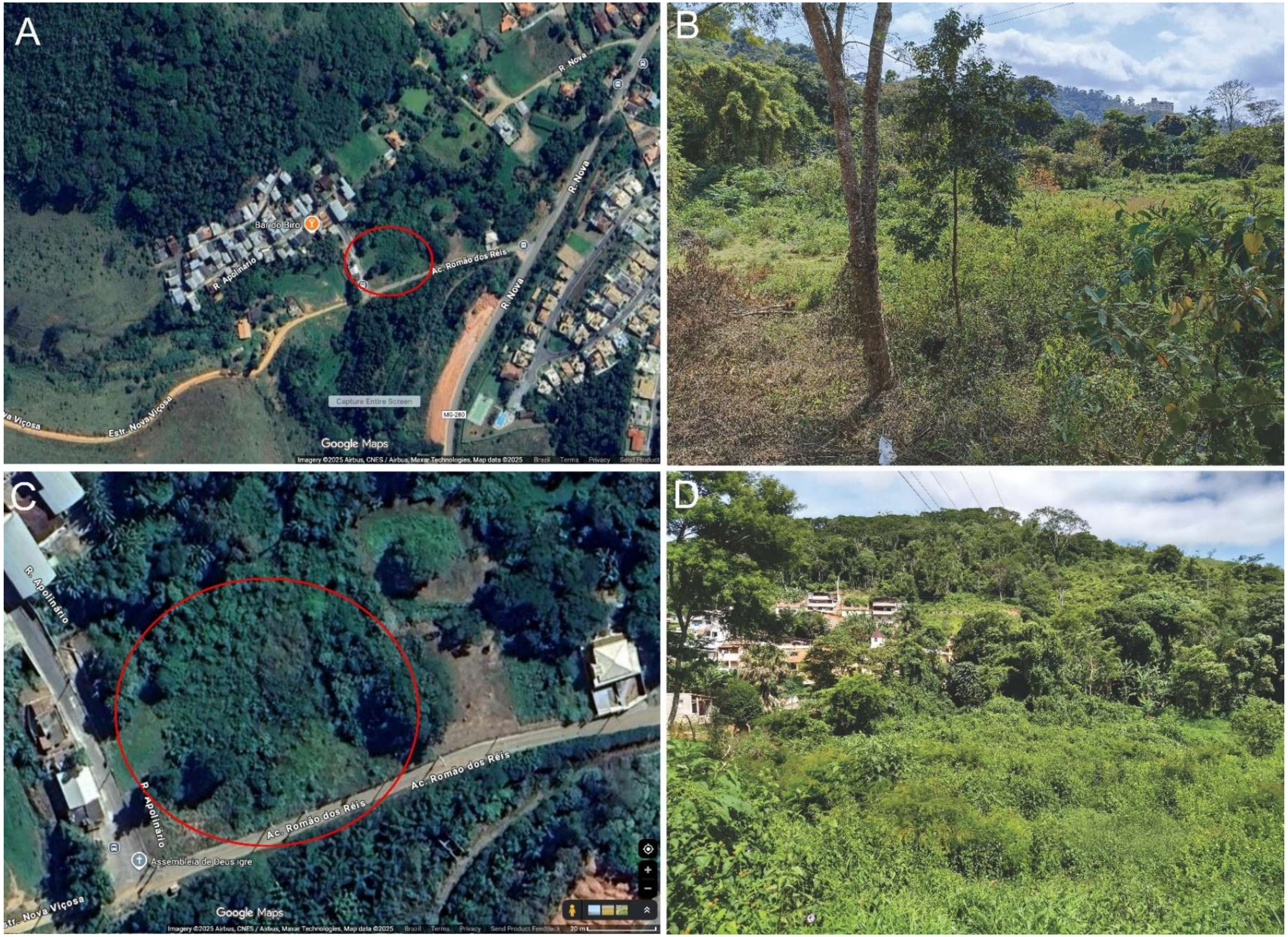

Suppl. Figure S2. Aspects of the area near Viçosa, MG, Brazil, where *Sida acuta* samples were collected from 2011 to 2022. **A, B.** Satellite images of the area and the surrounding region. The red circles indicate the area where samples were collected. Images from Google Maps, ©2025. **C, D.** General aspect of the area where samples were collected. Images obtained by the authors in 2024.

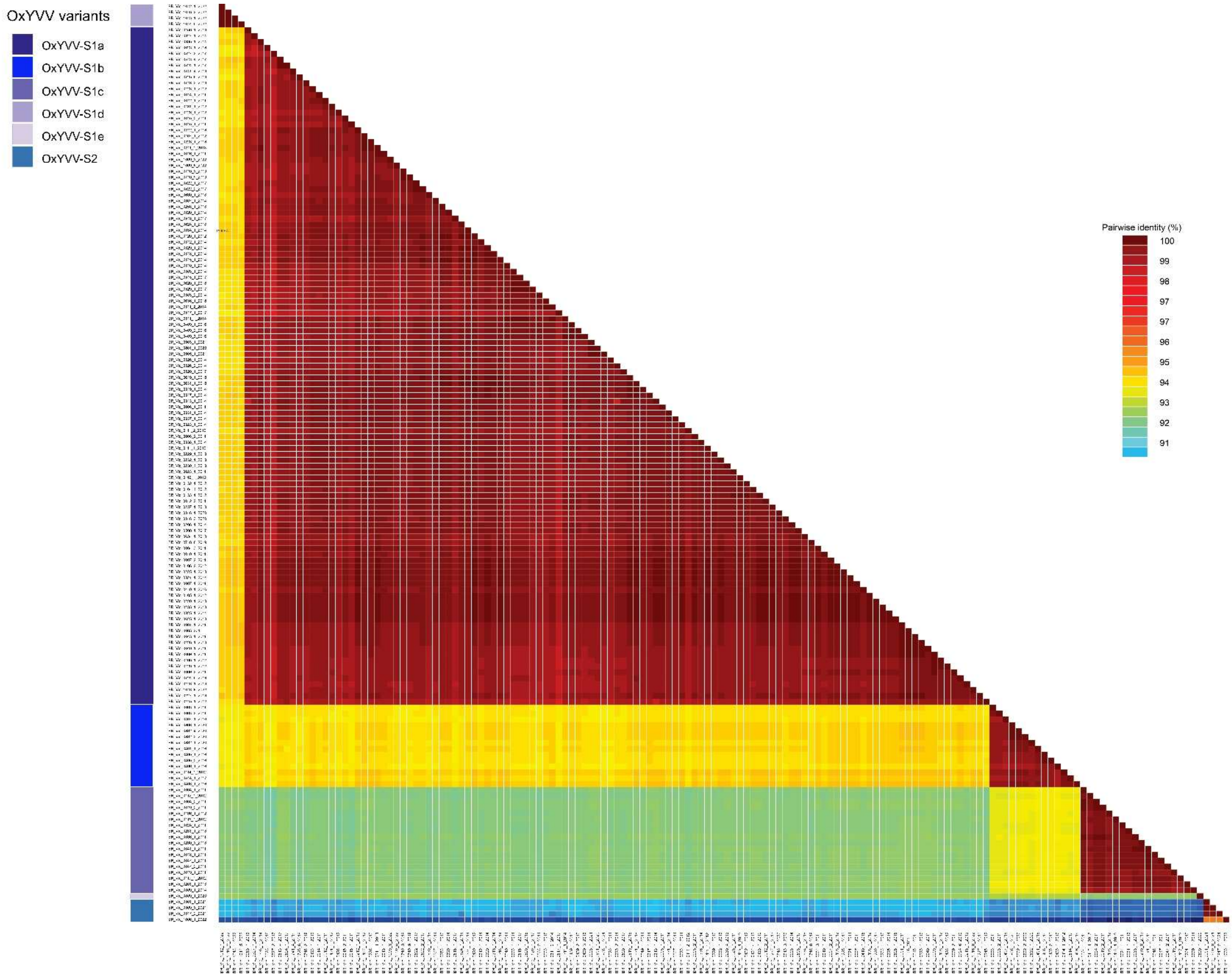

Suppl. Figure S3. Pairwise nucleotide sequence identity matrix of Oxalys yellow vein virus (OxYVV) isolates obtained in this work. The colored bar at the left indicates the two strains and five variants identified based on the cut-off values determined by the *Geminiviridae* Study Group of the International Committee on Taxonomy of Viruses (ICTV) (Brown et al., 2015).

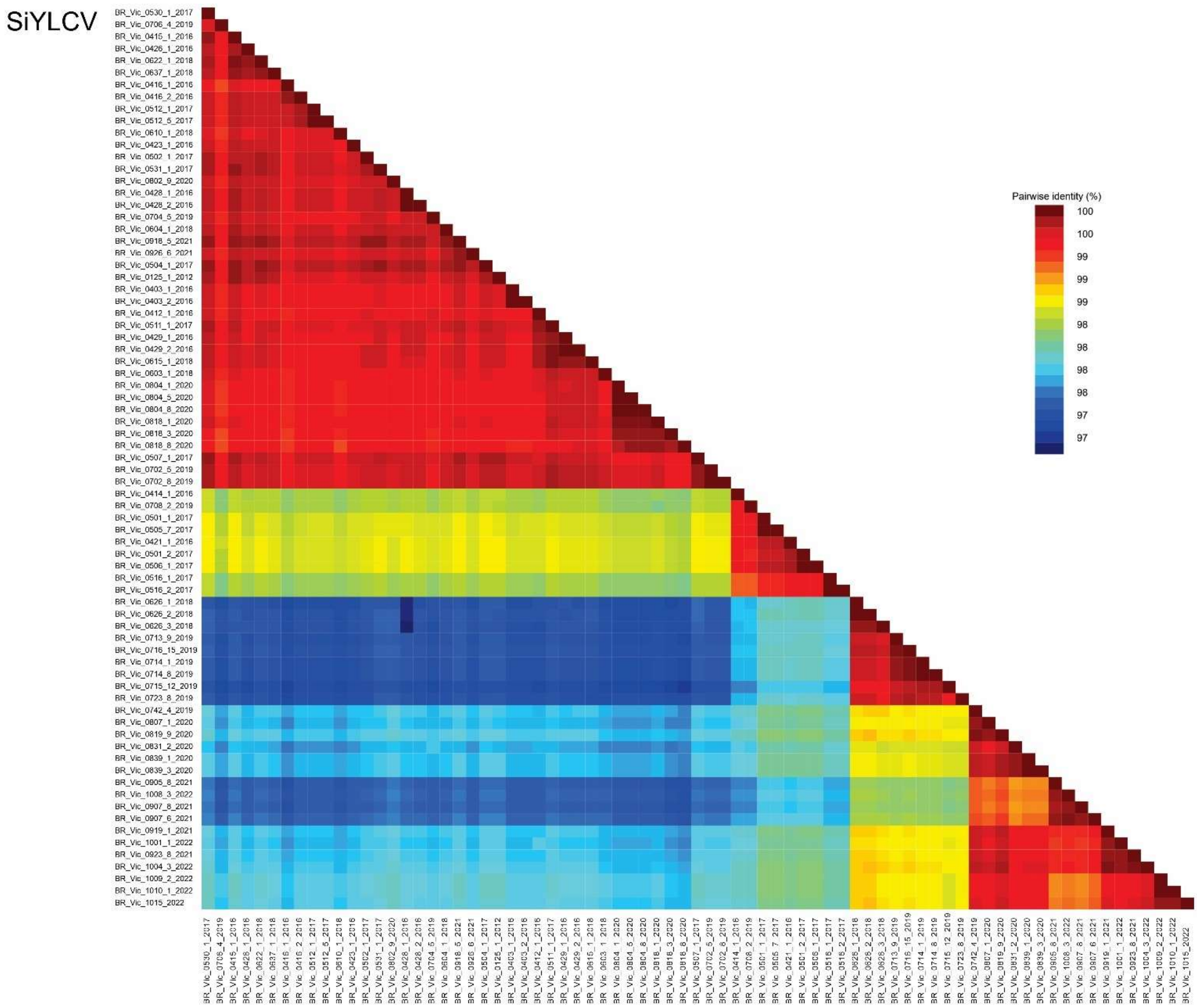

Suppl. Figure S4. Pairwise nucleotide sequence identity matrix of Sida yellow leaf curl virus (SiYLCV) isolates obtained in this work.

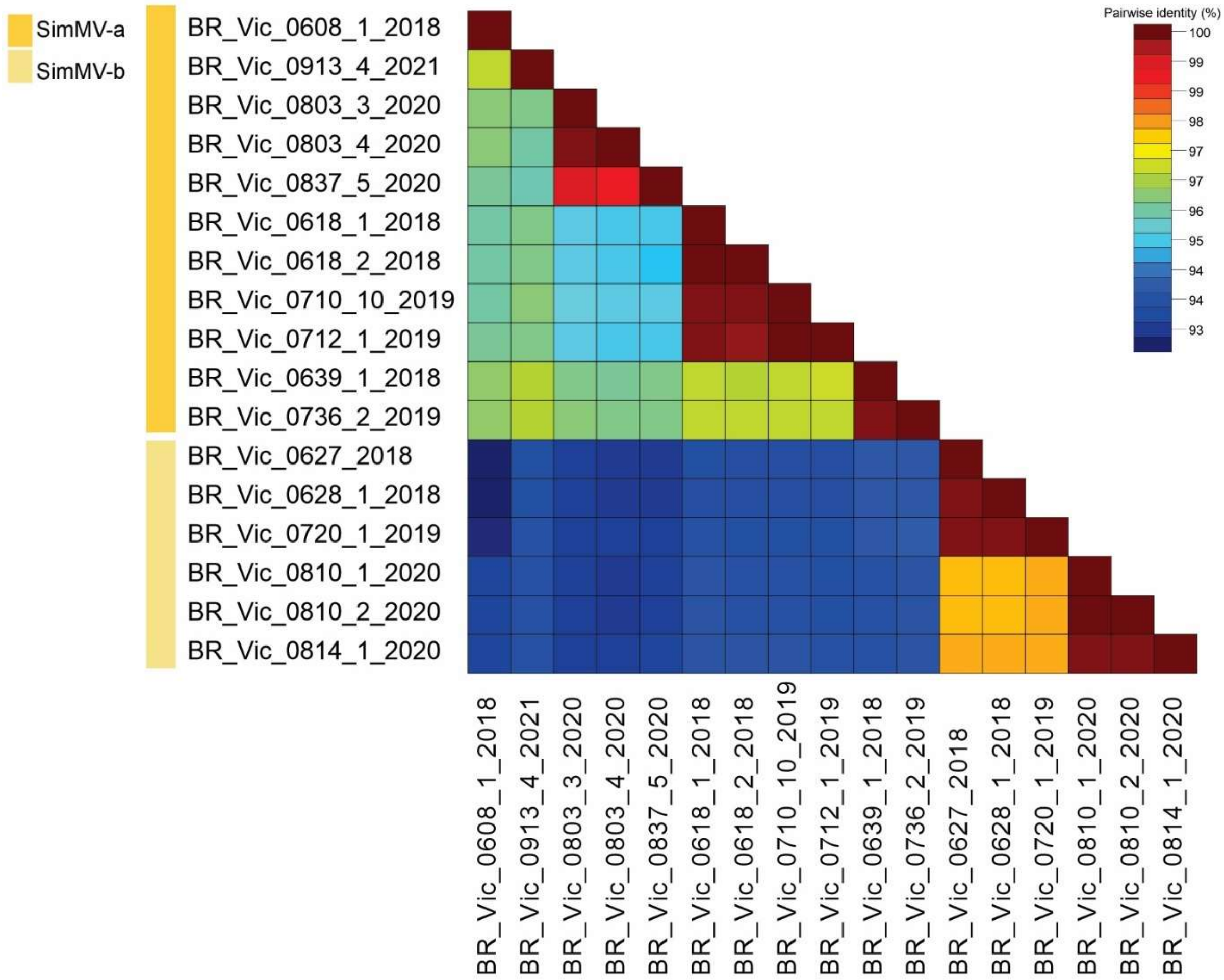

Suppl. Figure S5. Pairwise nucleotide sequence identity matrix of Sida micrantha mosaic virus (SimMV) isolates obtained in this work. The colored bar at the left indicates the two strains identified based on the cut-off values determined by the *Geminiviridae* Study Group of the International Committee on Taxonomy of Viruses (ICTV) (Brown et al., 2015).

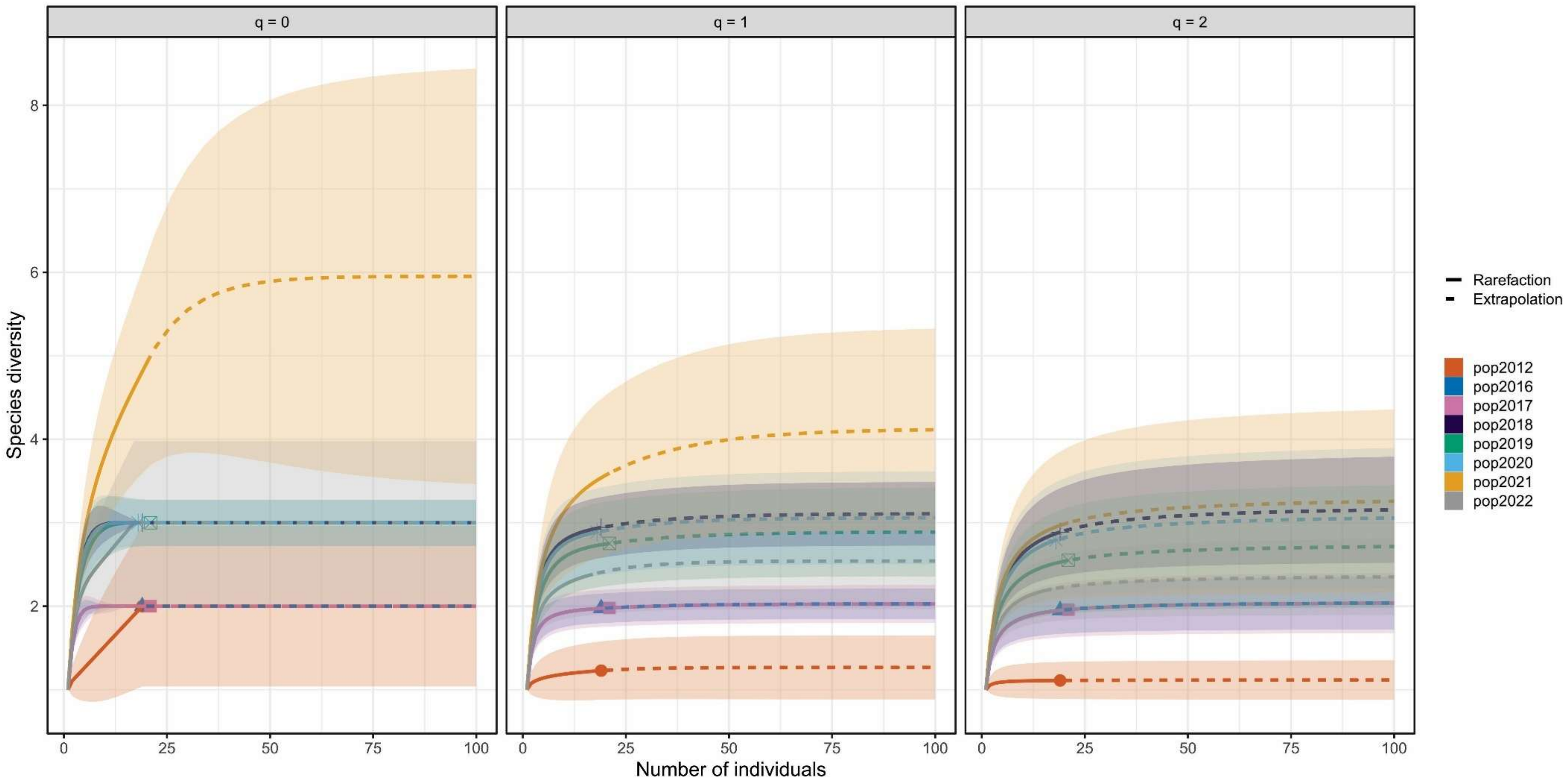

Suppl. Figure S6. Diversity accumulation curves for different sample sizes. The shaded background represents 95% confidence intervals of Hill numbers calculated for the three diversity orders (0, 1 and 2) estimated for each year of collection.

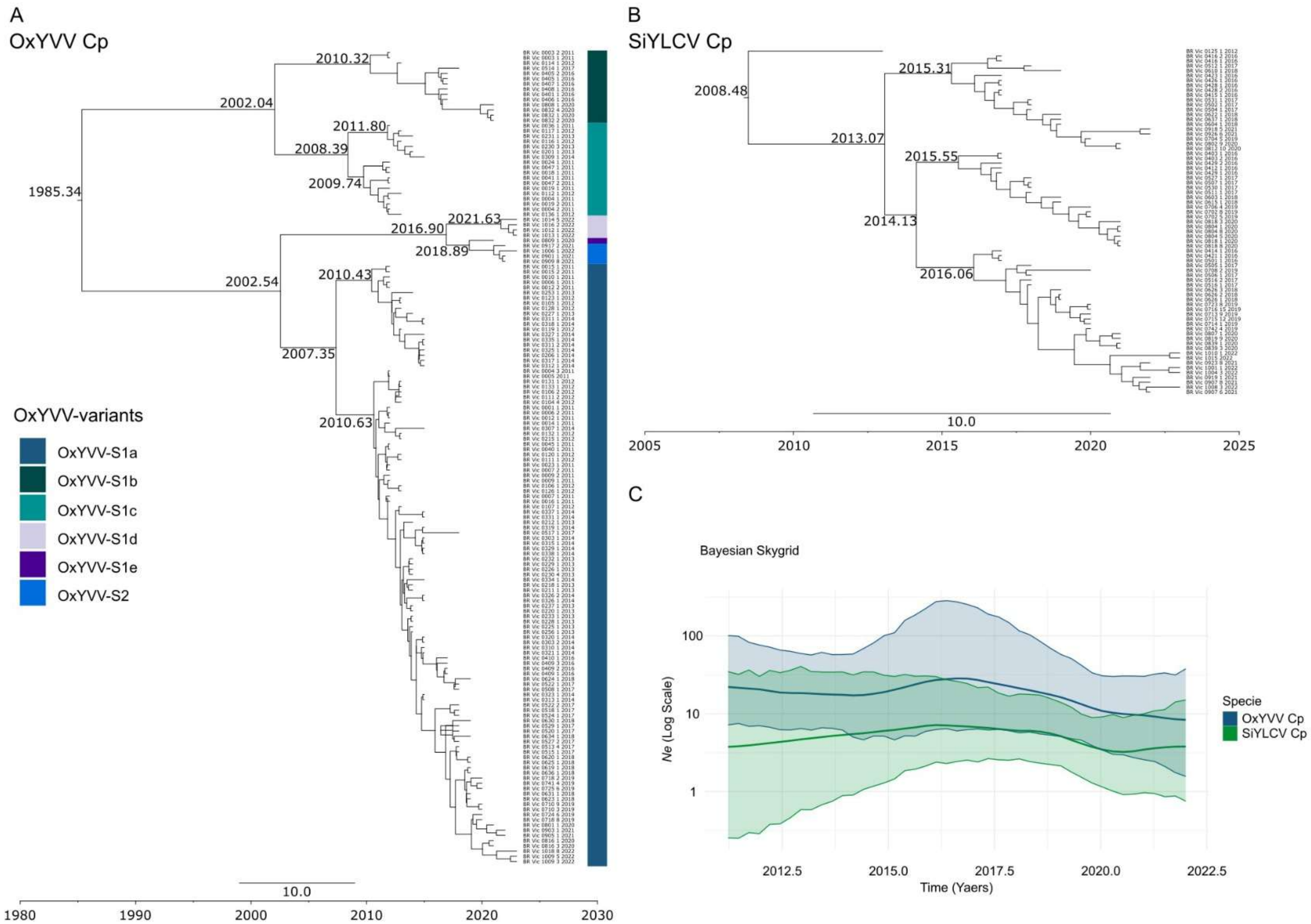

Suppl. Figure S7. Evolutionary and demographic dynamics of the Oxalis yellow vein virus (OxYVV) and Sida yellow leaf curl virus (SiYLCV) coat protein (*CP*) genes. **A.** Maximum clade credibility (MCC) phylogenetic tree for the OxYVV *CP* gene. The numbers on the branches indicate the time to the most recent common ancestor. The identified OxYVV variants (S1a-S1e and S2) are shown in distinct colors in the side bar. **B.** MCC tree for the SiYLCV *CP* gene. **C.** Demographic history under a coalescent framework (effective population size, Ne) inferred using the Bayesian Skygrid model for the OxYVV and SiYLCV *CP* genes. The lines represent the median estimates, and the shaded areas correspond to the 95% highest posterior density (HPD) intervals.

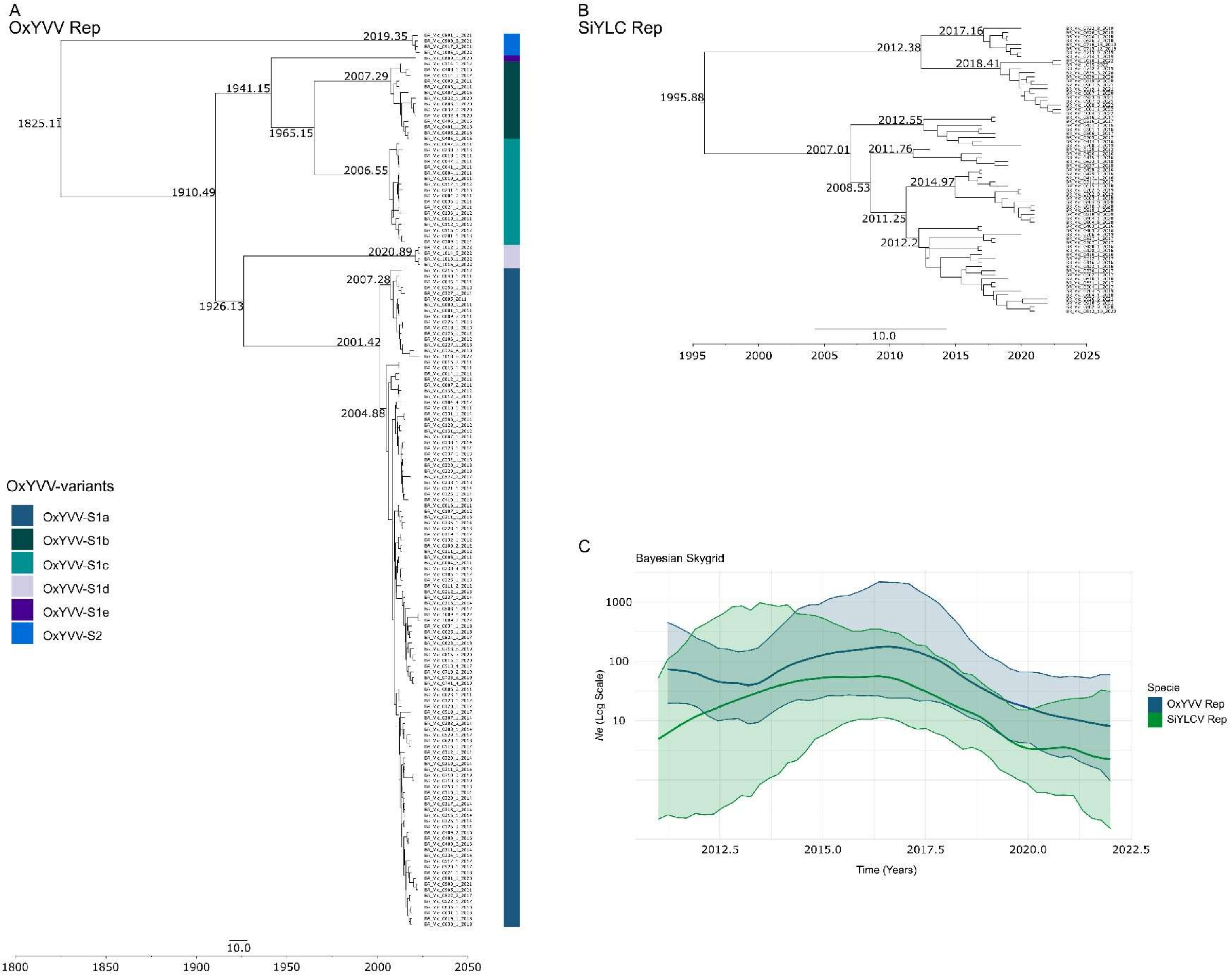

Suppl. Figure S8. Evolutionary and demographic dynamics of the Oxalis yellow vein virus (OxYVV) and Sida yellow leaf curl virus (SiYLCV) replication-associated protein (*Rep*) genes. **A.** Maximum clade credibility (MCC) phylogenetic tree for the OxYVV *Rep* gene. The numbers on the branches indicate the time to the most recent common ancestor. The identified OxYVV variants (S1a–S1e and S2) are shown in distinct colors in the side bar. **B.** MCC tree for the SiYLCV *Rep* gene. **C.** Demographic history under a coalescent framework (effective population size, Ne) inferred using the Bayesian Skygrid model for OxYVV and SiYLCV *Rep* genes. The lines represent the median estimates, and the shaded areas correspond to the 95% highest posterior density (HPD) intervals.
